# New Tricholomalides D–G from the Mushroom *Tricholoma ustaloides* Grown in an Italian Beech Wood

**DOI:** 10.3390/molecules28217446

**Published:** 2023-11-06

**Authors:** Gianluca Gilardoni, Francesca Negri, Paola Vita Finzi, Faiq H. S. Hussain, Giovanni Vidari

**Affiliations:** 1 Departamento de Química, Universidad Técnica Particular de Loja (UTPL), Loja 110107, Ecuador; ggilardoni@utpl.edu.ec; 2 Dipartimento di Chimica, Università degli Studi di Pavia, Via Taramelli 10, 27100 Pavia, Italy; f.negri@mail.com (F.N.); paola.vitafinzi@unipv.it (P.V.F.); 3 Department of Medical Analysis, Faculty of Applied Science, Tishk International University, Erbil 44001, Iraq; faiq.hussain@tiu.edu.iq

**Keywords:** *Tricholoma ustaloides*, *Tricholomataceae* (*Basidiomycota*), seconeodolastane diterpenoids, tricholomalides, CD spectra, absolute configuration

## Abstract

Four novel seconeodolastane diterpenoids, named tricholomalides D–G, were isolated, together with the known tricholomalide C, from the fruiting bodies of *Tricholoma ustaloides* Romagn., a species belonging to the large *Tricholoma* genus of higher mushrooms (*Basidiomycota*, family *Tricholomataceae*). They were isolated through multiple chromatographic separations, and the structures, including the absolute configuration, were established through a detailed analysis of MS, NMR, and CD spectral data and comparison with related compounds reported in the literature, which has been thoroughly revised.

## 1. Introduction

More than 250 species are included in the genus *Tricholoma* (Fr.) Staude [1], which is the largest in the family *Tricholomataceae* of the order *Agaricales* (*Basidiomycota*). The species have a worldwide distribution and are mainly found in temperate and subtropical zones in both the southern and northern hemispheres [2], from Australia and China to North America and Europe [3,4,5,6,7,8].

The use of molecular methods based on nuclear rDNA internal transcribed spacer ITS1-5.8S-ITS2 (ITS) sequences is becoming more and more important in phylogenetic studies of higher mushrooms [7,8,9], accompanying or even substituting the traditional studies of fungal morphological characters. Thus, it has often been demonstrated that mushrooms growing in different habitats/countries but classified as the same species based on morphological characters, must instead be placed in distinct clades/subclades or even different taxa [8,10,11]. In this context, the distinct chemical contents determined through phytochemical analysis have often confirmed the possible existence of different varieties or taxa [12]. 

Our interest in the chemical analysis of *Tricholoma* species grown in Italy was thus motivated by taxonomical reasons, as the morphological characters alone make it difficult to differentiate some species, e.g., those included in the section *Genuina* [7]. However, not less important was the fact that the fruiting bodies of most *Tricholoma* produce a wide variety of secondary metabolites, exhibiting unusual/rare structures and important biological activities, including cytotoxic and antimicrobial properties, neurite outgrowth stimulation effects, acetylcholinesterase inhibitory activity, etc. [13].

**Figure 1:**
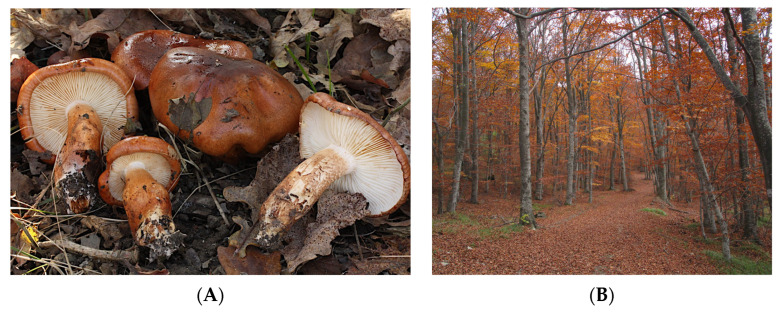
Specimens of *Tricholoma ustaloides* (**A**) collected in a beech wood (**B**) in Northern Apennines near Pavia, Italy (photos provided by Teresio Restelli and Alfredo Gatti).

Continuing our studies on *Tricholoma* species grown in Italy, our attention was recently drawn to the phytochemical study of *Tricholoma ustaloides* Romagn. (Figure 1A), which has been included by Heilmann-Clausen and Christensen in the critical section *Genuina* [7]. This mushroom, which is considered inedible in Europe, is generally rare in Italy, where it typically grows from late summer to late autumn in association with oak, chestnut, hornbeam, and beech trees [4,5] (Figure 1B).

**Figure 2:**
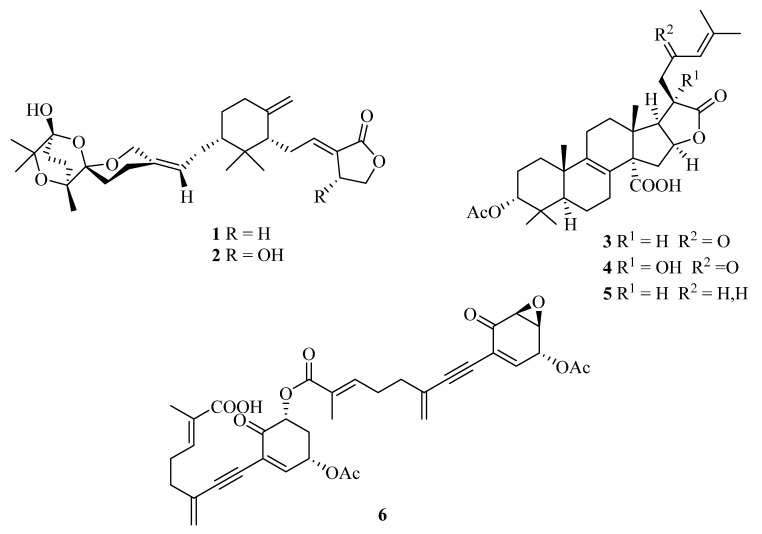
Structures of saponaceolides J (**1**) and F (**2**), tricholidic acid (**3**), tricholidic acids B (**4**) and C (**5**), and tricholomenyn C (**6**) isolated from *Tricholoma ustaloides*.

In our initial phytochemical investigation of an aqueous methanol sub-extract of an EtOAc extract of *T. ustaloides* fruiting bodies, we isolated two rare C-30 terpenoids, saponaceolides J (**1**) and F (**2**), three cyclic lactone-containing lanostane triterpenoids, tricholidic acid (**3**), the new tricholidic acids B (**4**) and C (**5**), and the rare tricholomenyn C (**6**) (Figure 2). In addition, mixtures of undetermined triglycerides and free fatty acids, together with five unidentified diterpenoids (A1–A5), were isolated [14].

In this paper we report the structures of compounds A1–A5, which were established through interpretation of IR, MS, 1D, and 2D NMR spectral data. The assignment of the absolute configuration (AC) to these compounds was based on the interpretation of electronic circular dichroism (ECD) spectra. In general, determination of the absolute configuration of natural products using ECD compares the spectrum of a new compound having an unknown AC to those of analogous compounds of known ACs [15]. However, AC determination by predicting the sign of one or more bands in the ECD spectrum using empirical, semiempirical, or nonempirical rules is often an option [15,16]. Another widely used option is to compare calculated and experimental ECD spectra [15].

The assignment of stereostructures to the compounds A1–A5 heavily depended on those of related diterpenoids previously isolated from other *Tricholoma* species. Since there are several discrepancies in the literature about the structures of the latter compounds [13], especially about their ACs, the discussion of the structures of A1–A5 is preceded by a short critical review of the existing literature.

## 2. Diterpenoids Isolated from *Tricholoma* Fruiting Bodies: A Short Critical Review

Diterpenoids are a class of terpenes formally composed of four isoprene units, that are biosynthesized by plants, animals, and fungi, including higher mushrooms (*Basidiomycota*) [17], via the HMG-CoA reductase pathway, with geranylgeranyl pyrophosphate being a primary intermediate [18]. Only a few examples of diterpenoids have been isolated so far from fruiting bodies of *Tricholoma* [13].

### 2.1. Trichoaurantianolides and Tricholomalides

**Scheme 1:**
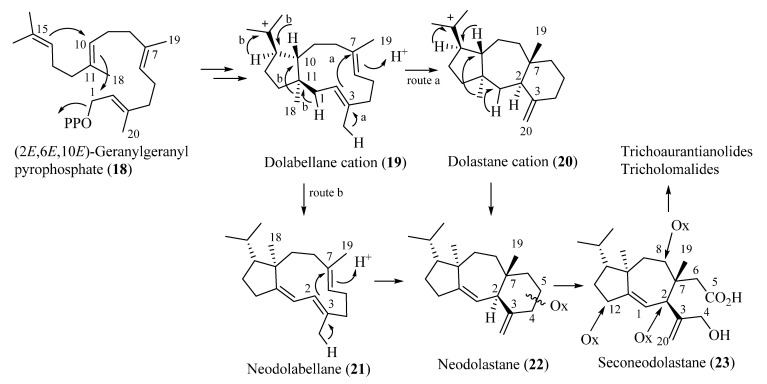
Proposed biosynthetic pathways of trichoaurantianolides and tricholomalides.

All the diterpenoids isolated from fruiting bodies of *Tricholoma* species have a rare rearranged terpenoid skeleton, which has been named seconeodolastane in accordance with the proposed biosynthetic pathway (vide infra, Scheme 1). They belong to two diastereomeric families, the trichoaurantianolides and the tricholomalides, respectively, depending on the stereochemistry of the OH group at the C-8 position of the seconeodolastane skeleton (vide infra).

The first group of diterpenoids includes trichoaurantianolides A–D (**7**–**10**), which were isolated from wild fruiting bodies of *T. aurantium* (Schaeff.: Fr.) Ricken collected in Italy [19,20]; trichaurantin **8** (*synonym* of trichoaurantianolide B), which was isolated from the fruiting bodies of *T. aurantium* and *T. fracticum* (Britz.) Kreis. (syn. *T. batschii* Gulden) collected in Germany [21]; and acetyl trichaurantin (**11**), which was isolated from *T. aurantium* [21].

**Figure 3:**
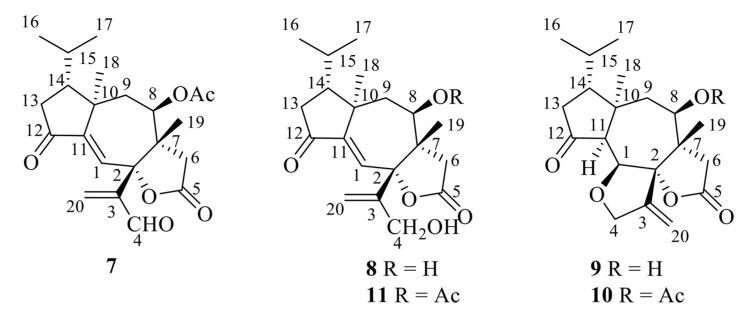
Structures of trichoaurantianolides A–D (**7**–**10**) and acetyl trichaurantin (**11**).

**Figure 4:**
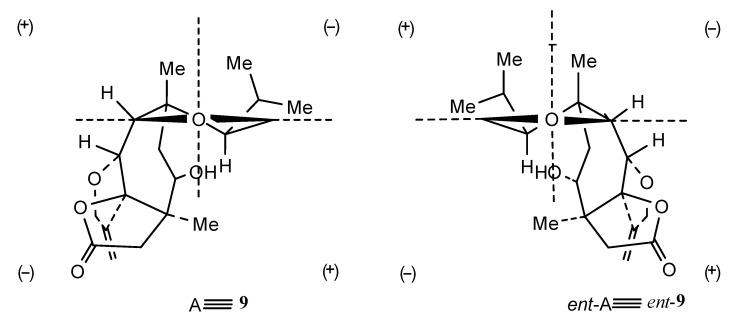
A: octan projections of the “twisted” conformation of the cyclopentanone ring in trichoaurantianolide C (**9**); B: octan projection of *ent*-**9**.

Structures **7**–**11** were established using 2D NMR data and using single-crystal X-ray analysis in the case of alcohol **8** [20,21]. Moreover, the absolute configurations of trichoaurantianolides B (**8**), C (**9**), and D (**10**) (Figure 3) were firmly established using anomalous X-ray diffraction and chemical interconversions [20,21], and enantioselective total synthesis [22]. Thus, the absolute stereochemistry of trichoaurantianolide C (**9**) was identical to the one initially assigned to this compound on the basis of the weak Cotton effect (CE) observed at 300 nm (Δε = +0.14) [20]. In fact, based on an empirical rule [16], the positive sign of the CE of compound **9**, for which the molecular modeling indicated a “twisted” conformation of the cyclopentanone ring, corresponded to the signs of the octants occupied by the carbons of the ring (see structure A in Figure 4). Instead, misapplication of the octant rule [16] to the positive CE of trichoaurantianolide C resulted in the erroneous assignment of the enantiomeric configuration *ent*-**9** (see structure *ent*-A in Figure 4) [23].

**Figure 5:**
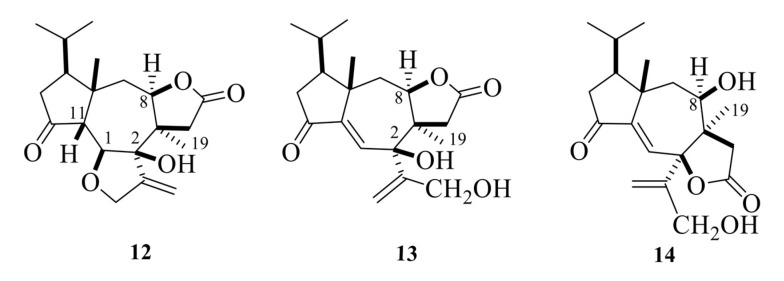
Structures of tricholomalides A–C [23,24].

Tricholomalides A–C were isolated from the methanol extract of fresh fruiting bodies of an undetermined species of *Tricholoma* collected in Japan [23]. Noteworthily, these diterpenoids significantly induced neurite outgrowth in rat pheochromocytoma cells at concentrations of 100 μM [23]. The stereostructures of tricholomalides A–C, which were similar to those of trichoaurantianolides **7**–**11**, were definitely established as **12**–**14** (Figure 5) using total synthesis and single-crystal X-ray diffraction. Thus, the stereochemistry at C-2 in tricholomalides A (**12**) and B (**13**) [24] was established to be opposite to the one originally assigned to these compounds based on spectral data only [23]. Moreover, H-8 was *cis* to H_3_-19, while it was *trans* in compounds **7**–**11**.

The absolute configuration of tricholomalides A–C has not yet been determined unambiguously. However, biosynthetic considerations (vide infra, Scheme 1) and interpretation of the CD data applying the empirical rule of twisted cyclopentanones [16], instead of the octant rule used in the paper reporting the isolation of tricholomalides [23], have strongly suggested that the configuration is identical to that of trichoaurantianolides, thus inverting the original assignment [23]. In fact, like trichoaurantianolide C (**9**) [20], tricholomalide A (**15**) exhibited a positive Cotton effect at 302 nm (Δε + 0.31) [23], indicating a similar “positively twisted” conformation of the cyclopentanone ring (see the discussion above). In addition, tricholomalide B (**16**) was converted to **15** during storage at 4 °C in DMSO [23], and the CD spectra of the *cisoid* α,β-unsaturated ketones **16** and **17** revealed CEs with the same signs for the corresponding peaks at 250 (Δε − 0.57) and 336 nm (Δε + 0.53) for **16** and at 234 (Δε − 0.22) and 344 nm (Δε + 0.16) for **17** [23].

**Figure 6:**
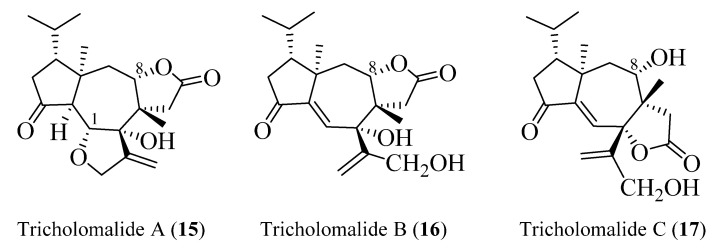
Absolute configurations proposed for tricholomalides A–C.

In summary, the absolute stereochemistry depicted in Figure 6 is proposed for tricholomalides A–C (**15**–**17**).

Biosynthetically, trichoaurantianolides and tricholomalides should originate from an enzymatically controlled stereoselective cyclization of geranylgeranyl pyrophosphate (**18**) producing nonracemic dolabellane cation **19**. This intermediate would be the precursor of the dolastane (**20**), neodolabellane (**21**), and neodolastane (guanacastane) (**22**) skeletons through a series of stereospecific transannular cyclizations and Wagner–Meerwein migrations (Scheme 1). The seconeodolastane skeleton (**23**) of trichoaurantianolides and tricholomalides would finally result from oxidation and cleavage of the six-membered ring in **22** (Scheme 1), while oxidative decoration of the structures at C-2, C-8, and C-12 would likely occur in the latest steps of the biosynthetic path to trichoaurantianolides and tricholomalides. It is interesting to note that diterpenoids originating from a common biogenetic pathway in algae, fungi, liverworts, and higher plants are enantiomeric to the corresponding metabolites isolated from species of marine invertebrates [13,25].

## 3. Results and Discussion

### 3.1. Tricholomalides from Tricholoma Ustaloides

**Figure 7:**
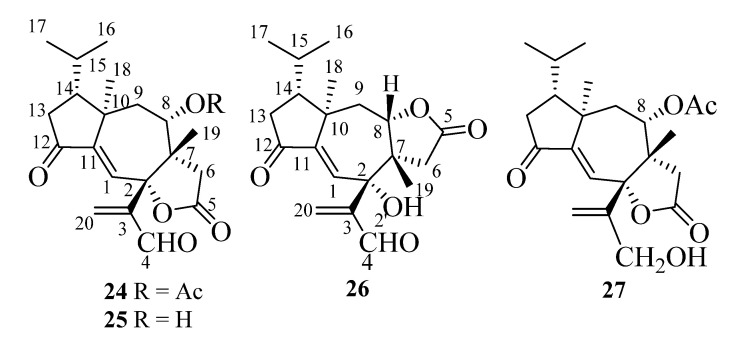
Structures of tricholomalides D–G (**24**–**27**).

In the following paragraphs, we will describe the determination of the structures of compounds A1–A5, whose isolation from *T. ustaloides* fruiting bodies was reported in a recent paper [14]. A1–A4 were new diterpenoids, named tricholomalides D–G (**24**–**27** in Figure 7), respectively, while A5 was identified as the known tricholomalide C (**17**) [23]. At first, we will discuss the structures of tricholomalides D (**24**), E (**25**), and G (**27**), which showed a γ-lactone ring *cis*-fused at C-2 and C-7 with the central cycloheptene ring of the seconeodolastane skeleton. Subsequently, we will describe the structure of tricholomalide F (**26**), in which the γ-lactone ring is *cis*-fused with the cycloheptene ring at C-7 and C-8.

#### 3.1.1. Tricholomalides C, D, E, and G

The molecular formula of tricholomalide D (**24**) was established as C_22_H_28_O_6_ by EIMS (Figure S9 in the Supplementary Materials), elemental analysis, and ^1^H and ^13^C NMR spectra (Table S1 in the Supplementary Materials). It indicated nine degrees of unsaturation. The oxygen-containing functionalities in the molecule were established as one cyclopentanone (C-12), one formyl (C-4), one γ-lactone (C-5), and one acetoxy (C-21) group from the IR bands (1783, 1736, 1699 cm^−1^) and the characteristic chemical shifts (*δ*_C_ 205.0, 191.8, 175.2, and 169.4 ppm, respectively) of the corresponding carbonyl carbons in the ^13^C NMR spectrum (Figures S7 and S8 in the Supplementary Materials).

**Figure 8:**
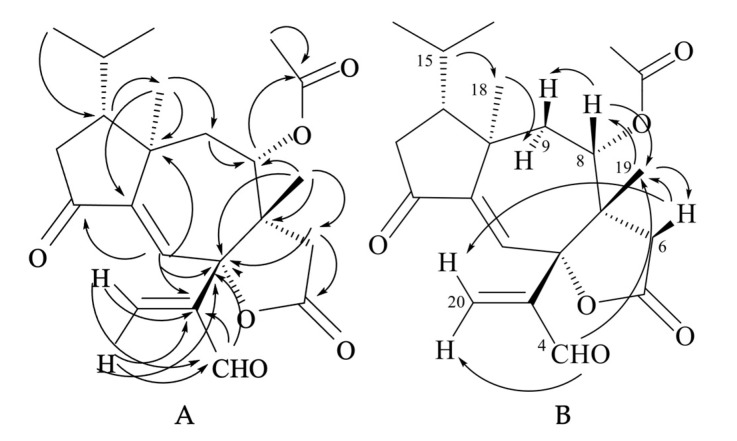
HMBC (H → C) correlations (**A**) and 1D NOE correlations (H → H) (**B**) observed for tricholomalide D (**24**).

The NMR spectra of compound **24** (Figures S5 and S7 and Table S1 in the Supplementary Materials) showed signals for one trisubstituted (*δ*_C_ 125.9 (d, C-1) and 151.3 (s, C-11); *δ*_H_ 6.63 (1H, s, H-1)) and one *exo*-methylene double bond (*δ*_C_ 138.4 (t, C-20) and 148.6 (s, C-3); *δ*_H_ 6.41 (1H, br s, H*_Z_*-20) and 6.62 (1H, br s, H*_E_*-20)). HMBC correlations (Figure 8A) indicated that the two olefins were α,β-conjugated to the ketone (C-12) and the aldehyde (C-4) groups, respectively, while they were bound to each other through an oxygenated quaternary carbon placed in the lactone ring (*δ*_C_ 87.4 (s, C-2)). The remaining two unsaturations of tricholomalide D (**24**) were then assigned to two rings that formed a tricyclic carbon skeleton together with the γ-lactone. The ^1^H NMR spectrum of **24** (Figure S5 in the Supplementary Materials) showed two methyl singlets at *δ*_H_ 0.98 (H_3_-19) and 1.12 (H_3_-18) that were attached to quaternary carbons C-7 (*δ*_C_ 49.8 s) and C-10 (*δ*_C_ 44.7 s), respectively, and two methyl doublets of an isopropyl group at *δ*_H_ 0.95 (*J* = 6.5 Hz, H_3_-17) and 1.02 (*J* = 6.5 Hz, H_3_-16). The remaining proton and carbon atoms of compound **24** were assigned to three separate moieties, I–III based on ^1^H-^1^H COSY data (Figure S6 in the Supplementary Materials) and selective homonuclear decoupling experiments. The moiety I linked C-13 to C-16 and included the H-15 (*δ*_H_ 1.75–1.85, 1H, m) to H_3_-17 linkage. The spin system of the unit II was an isolated AB quartet (*δ*_H_ 2.29 and 2.88, 2H, *J*_AB_ = 18.5 Hz), which was assigned to an isolated methylene (H_2_-6) attached to the γ-lactone carbonyl group (*δ*_C_ 175.2, s, C-5) by HMBC correlations (Figure 8A). The moiety III gave rise to a distorted AMX system which includes a methylene (H_2_-9; *δ*_H_ 1.79 (1H, dd, 15.0, 11.5) and *δ*_H_ 2.15 (1H, dd, 15.0, 2.0)) and a methine hydrogen (H-8; *δ*_H_ 4.72 (1H, ddd, 11.5, 2.0, 1.0)). This proton was geminal to the acetoxy carbonyl carbon (*δ*_C_ 169.4, s, C-21) via an HMBC cross peak with C-21 (Figure 8A). Assembling these partial structures using the two- and three-bond C-H connectivity data from HMBC correlations (Figure 8A), we unambiguously assigned structure **24** to tricholomalide D. The structure of the ring system was determined by (i) the bonds around the angular methyl (H_3_-19) attached to C-7 (*δ*_C_ 49.8 s), which linked C-6 (*δ*_C_ 35.3 t) and C-9 (*δ*_C_ 37.7 t) to C-19 (*δ*_C_ 22.4 q) via C-7 and C-8 (*δ*_C_ 74.4 d); (ii) the linkages from H_3_-19, H_2_-20, and H-4 (*δ*_H_ 9.70 (1H, s)) to C-2 (*δ*_C_ 87.4 s) and those from H-1 (*δ*_H_ 6.63 (1H, s)) to C-2, thanks to which the acrolein moiety was connected to C-2 and the arrangement of the substituents of the γ-lactone ring was defined; (iii) the connectivities from H_3_-18 to C-10 (*δ*_C_ 44.7 s), C-9 and C-11 (*δ*_C_ 151.3 s), and H-1 to C-10, of which C-12 and C-2 indicated the presence of a cycloheptene ring bearing an angular methyl (H_3_-18) on an allylic sp^3^ quaternary carbon (C-10); (iv) the linkage of the isopropyl group to C-18 (*δ*_C_ 21.4 q) via C-14 (*δ*_C_ 47.2 d), which defined the placement of the moiety I to form a cyclopentanone ring. These data and 1D NOE correlations (Figure 8B), H-15/H_3_-18; H_3_-18/H-9α; H-8/H-9β and H-8/H_3_-19; H_3_-19/H-6β, H-4/H_3_-19, and H-6β/H-20*E* (Figure 8B), suggested that tricholomalide D (**24**) was the C-8 epimer of trichoaurantianolide A (**7**) [19]. Thus, the 8-acetoxy group was *trans* to H_3_-19 in **24**, while it was *cis* in **7** [19]. This finding was confirmed by the significantly different chemical shift and coupling constants of H-8 in **24** (*δ*_H_ 4.72; *J*_8β-9β_ = 2.0 and *J*_8β-9α_ = 11.5 Hz) and in **7** (*δ*_H_ 5.15; *J*_8α-9β_ = 9.5 and *J*_8α-9α_ = 1.5 Hz) [19].

The molecular formula of tricholomalide E (**25**), C_20_H_26_O_5_, was established from the EIMS (Figure S15 in the Supplementary Materials) and the ^1^H and ^13^C NMR spectra (Figures S11–S14 in the Supplementary Materials). The NMR spectral data (Table S1 in the Supplementary Materials) clearly indicated that it was the deacetyl derivative of tricholomalide D (**24**). In fact, the signals of an acetyl group were missing in the ^1^H and ^13^C NMR spectra of **25** (Figures S11 and S13 in the Supplementary Materials), while the signals of H-8 (*δ*_H_ 3.63, br d, 11.5) and C-8 (*δ*_C_ 71.6 d) in the ^1^H and ^13^C NMR spectra, respectively, of alcohol **25** moved upfield by about 1 and 3 ppm, respectively, in comparison with the corresponding signals of the acetate **24** (vide supra). Finally, the structure of **25** was confirmed with standard acetylation with Ac_2_O/Py, which afforded a product that was identical with acetate **24**.

Tricholomalide G (**27**), with the molecular formula C_20_H_26_O_5_ from the EIMS (Figure S27 in the Supplementary Materials) and the ^1^H and ^13^C NMR spectra (Figures S23–S26 in the Supplementary Materials), was identified as the dihydroderivative of aldehyde **24** through the following significant differences between the NMR spectra of the two compounds (Figures S23–S26 and Table S1 in the Supplementary Materials): (i) the presence of two diastereotopic protons (H_2_-4) at *δ*_H_ 4.22 (1H, dd, 13.0, 1.1) and 4.27 (1H, br d, 13) in the ^1^H NMR spectrum of **27**, assignable to an allylic C*H*_2_OH group, which replaced the signal H-4 (*δ*_H_ 9.70 (1H, s)) of aldehyde **24**; (ii) the upfield shift of the H_2_-20 protons (*δ*_H_ 5.32 (1H, s) and *δ*_H_ 5.67 (1H, t, 1.1)) and the C-20 carbon (*δ*_C_ 118.3, t), respectively, in the ^1^H and ^13^C NMR spectra of **27**, compared with the corresponding signals of the βCH_2_ group of the acrolein moiety in **24**. Finally, the structure of compound **27** was confirmed through the oxidation of the allylic alcohol with PDC (pyridinium dichromate), which afforded a product indistinguishable from aldehyde **24**.

The NMR spectral data (Figures S1–S3 in the Supplementary Materials) and the signs of the CD maxima of compound A5 (Figure S4 in the Supplementary Materials) were identical with the data reported in the literature for tricholomalide C (**17**) [23]. Moreover, selective oxidation of A5 (≡**17**) with PDC gave a product indistinguishable from aldehyde **25**.

#### 3.1.2. Tricholomalide F

Tricholomalide F (**26**) possessed the same molecular formula as tricholomalide E (**25**), C_20_H_26_O_5_, as inferred from the EIMS spectrum (Figure S21 in the Supplementary Materials) and the ^1^H and ^13^C NMR spectral data (Figures S17–S20 and Table S2 in the Supplementary Materials). These data resembled those reported for tricholomalide B (**16**) [23], except for the presence of a formyl group (H_4_ (*δ*_H_ 9.55, 1H, s) and a C-4 carbonyl group (*δ*_C_ 196.3, d)) bound to C-3 (*δ*_C_ 148.6 s) in **26** which replaced the allylic CH_2_OH group occurring in compound **16** [23]. The presence of a free tertiary OH group attached to C-2 of **26** was revealed by the singlet at *δ*_C_ 76.1 in the ^13^C NMR spectrum (Figure S19 in the Supplementary Materials), while the signals of H-8 (*δ*_H_ 3.92, 1H, dd, 12.8, 2.5) and C-8 (*δ*_C_ 85.0, d) in the NMR spectra of **26** (Figures S17 and S19 in the Supplementary Materials) indicated the ring closure of the γ-lactone unit on C-8.

**Figure 9:**
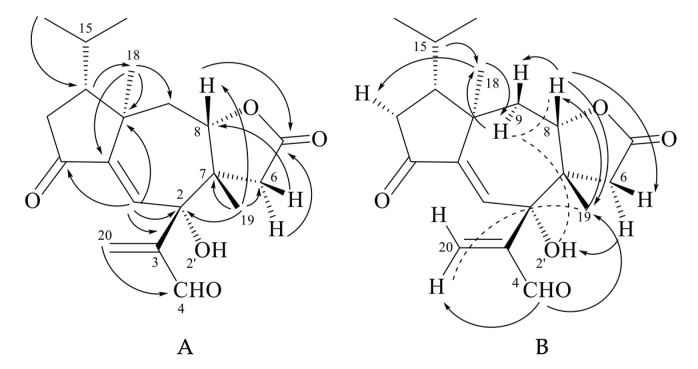
(**A**) HMBC (H → C) correlations; (**B**) 1D NOE correlations (H → H) observed for tricholomalide F (**26**); 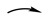 strong NOE effect; 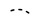 weak NOE effect.

The two- and three-bond HMBC correlations (Figure 9A) were fully consistent with the gross structure of **26**, while the relative stereochemistry of the stereogenic carbons in **26** was established through selective 1D NOE experiments (Figure 9B). In fact, the correlations of H-15 (*δ*_H_ 1.75–1.92 (1H, m)) with H_3_-18 (*δ*_H_ 1.20 (3H, s)) and between H_3_-18 and H-9α (*δ*_H_ 2.79 (1H, dd, 14.0, 12.8)) indicated that the isopropyl group, H_3_-18, and H-9α were oriented on the same side of the tricyclic structure, while the interactions of H-8 with H-9β (*δ*_H_ 2.23 (1H, dd, 14.0, 2.5)) and H_3_-19 (*δ*_H_ 0.97 (3H, br s)) and H-4 with H_3_-19 (Figure 9B) revealed that they were placed on the opposite sides of H_3_-18 and H-9α. The resulting stereochemistry of compound **26** was further corroborated by the *J* = 12.8 Hz of H-8β with H-9α and by the NOE interactions of H-8β with H-6β (*δ*_H_ 1.86 (1H, d, 17.0)) and H-6α (*δ*_H_ 3.24 (1H, dd, 17.0, 1.0)) with 2-O*H* (*δ*_H_ 4.92 (1H, d, 1.0)). Interestingly, H-1 (*δ*_H_ 6.97 (1H, d, 1.0)) was coupled with 2-O*H* by a *J*_W_ coupling of 1 Hz, which indicated a rather rigid orientation of the tertiary hydroxy group.

In summary, tricholomalides D–G (**24**–**27**) exhibited a relative stereochemistry identical to that of tricholomalides B (**16**) and C (**17**), while they were epimers of trichoaurantianolides at C-8.

#### 3.1.3. Absolute Configuration of Tricholomalides D–G

The absolute configuration of tricholomalides D–G (**24**–**27**) shown in Figure 7 was established on the basis of chemical interconversions, biosynthetic considerations (see above), and CD data (Figures S10, S16, S22 and S28 in the Supplementary Materials). The *cisoid* α,β-unsaturated cyclopentanone system occurring in **24**–**27** is also a characteristic feature of the structures of tricholomalides B (**16**) and C (**17**), as well as of trichoaurantianolides **7**, **8**, and **11**, whose configuration was thoroughly discussed in a previous Section 2.1. All the CD spectra of these compounds revealed Cotton effects with positive signs for the respective peaks in the region of 300–350 nm (Figures S10, S16, S22 and S28 in the Supplementary Materials), which were thus closely associated with similar conformational and dissymmetric substituent effects on the *cisoid* α,β-unsaturated ketone unit. Therefore, tricholomalides D–G (**24**–**27**) were assigned the same absolute configuration as tricholomalides B (**16**) and C (**17**) (Figure 6 and Figure 7), having a stereochemistry at C-8 that was opposite to that determined for trichoaurantianolides **7**, **8**, and **11** (Figure 3). This finding is not unexpected, as the configurations at C-7, C-10, and C-14 arise from stereospecific transannular cyclizations and Meerwein–Wagner rearrangements in the first steps of the common biosynthetic path leading to the tricholomalides and trichoaurantianolides (Scheme 1). Instead, the hydroxylation at C-8 likely involved a neodolastane or seconeodolastane derivative at a late step of the biosynthetic path, and it was controlled by enzymes with opposite diastereoselectivities for tricholomalides and trichoaurantianolides, respectively.

## 4. Materials and Methods

### 4.1. General Experimental Techniques and Procedures

Preparative chromatographic separations were carried out on open columns at atmospheric pressure (CC). The columns were manually packed with silica gel (Merck Kieselgel 60, 40–63 μm, Rahway, NJ, USA) or reversed-phase C_18_ (Merck LiChroprep RP-18, 25–40 μm) purchased from Sigma-Aldrich (St. Louis, MO, USA). Thin-layer chromatographic (TLC) analyses were conducted over glass-supported silica gel 60 (0.25 mm; GF_254_, Merck) or RP-18 (F254s, Merck) plates (Sigma-Aldrich). Spots on TLC plates were initially visualized under UV light (254 and 366 nm); subsequently, they were sprayed with a 0.5% solution of vanillin in H_2_SO_4_/ethanol 4:1 and finally heated with a hot gun until reaching maximum color development. Semipreparative medium-pressure liquid chromatographic (MPLC) separations were performed with an Isolera instrument (Biotage, Uppsala, Sweden) equipped with silica gel and RP-18 reversed-phase cartridges and a dual-wavelength UV detector. Reagent-grade solvents, purchased from Carlo Erba (Milan, Italy) or from Aldrich, were used for extraction and chromatographic separations. Optical rotation was conducted using PerkinElmer 241 polarimeter (Walthman, MA, USA); CD spectra were obtained using Jasco J-1500 CD spectrometer (Tokyo, Japan) in MeOH. IR spectra were obtained using PerkinElmer Paragon 100 PC FT-IR spectrometer (Walthman, MA, USA) on KBr disks. NMR spectra were obtained using Bruker AV300 and 400 spectrometers at 300 and 400 MHz (^1^H), respectively, and at 75.47 and 100 MHz (^13^C), respectively, operating at 22 °C (Billerica, MA, USA). ^1^H NMR and ^13^C NMR chemical shifts are relative to signals of residual CHCl_3_ (δ_H_ 7.25, singlet) and ^13^CDCl_3_ *δ*_C_ (77.0, central line of a triplet) in CDCl_3_ (Sigma-Aldrich, Steinheim, Germany); coupling constants (*J*) were measured in Hz; multiplicity (=number of attached hydrogens) of each C-atom was determined using DEPT experiments; COSY, DEPT, and HSQC spectra and NOE effects were recorded using standard pulse sequences. EIMS and DCI-MS (NH_3_) spectra were obtained using Finnigan-MAT 822 mass spectrometer.

### 4.2. Fungal Material

The fruiting bodies of *Tricholoma ustaloides* Romagn. were collected in a beech wood at the end of September—beginning of October 2021 and identified by Alfredo Gatti, as reported in the inaugural study of this mushroom [14]. A sample specimen (accession code: TU001) was deposited at the Department of Chemistry, University of Pavia, Italy.

### 4.3. Extraction and Isolation

Extraction of the fresh fruiting bodies (990 g) with EtOAc, evaporation of the extract, and partition of the resulting residue between MeOH-H_2_O at 90:10 and hexane were described in a previous paper [14]. The residue (2.8 g) from the aqueous methanol layer was subjected to multiple separations using semipreparative MPLC silica gel and RP-18 columns to afford compounds A1–A5, named tricholomalides D (**24**, 51 mg, 0.0051% on fresh fruiting bodies), E (**25**, 18.6 mg, 0.0019%), F (**26**, 5.5 mg, 0.0006%), G (**27**, 15.2 mg, 0.0015%), and C (**17**, 13.6 mg, 0.0014%), respectively [14].

#### 4.3.1. Tricholomalide C (**17**)

Colorless, sticky oil; [α]_D_^22^ + 32.9 (*c* 10.1 mg/mL, CH_2_Cl_2_); R_f_ 0.77 (RP18 TLC; MeOH-H_2_O, 6:1); CD (MeOH) Δε_236_ −0.61, Δε_345_ +0.51; ^l^H NMR (300 MHz, CDCl_3_) *δ*_H_ 0.98 (3H, d, 6.5, H_3_-17), 1.07 (3H, d, 6.5, H_3_-16), 1.14 (3H, s, H_3_-19), 1.16 (3H, s, H_3_-18), 1.68-1.89 (2H, m, H-14 and H-15), 1.84 (1H, dd, 15.5 and 11.5, H_α_-9), 2.11 (1H, dd, 15.5 and 2.0, H_β_-9), 2.14 (1H, dd, 18.5 and 12.5, H_α_-13), 2.37 (1H, dd, 18.0 and 1.0, H_β_-6), 2.47 (1H, dd, 18.5 and 8.0, Hβ-13), 2.84 (1H, d, 18.0, Hα-6), 3.48 (1H, ddd, 11.5, 2.0, and 1.0, H-8), 4.21 (2H, br s, H_2_-4), 5.30 (1H, s, H*_Z_*-20), 5.63 (IH, s, H*_E_*-20), 6.62 (1H, s, H-1); ^13^C NMR (75.5 MHz, CDCl_3_) *δ*_C_ 21.2 (q, C-18), 21.7 (q, C-17), 21.8 (q, C-19), 24.0 (q, C-16), 28.5 (d, C-15), 34.2 (t, C-6), 39.6 (t, C-9), 41.2 (t, C-13), 44.6 (s, C-10), 49.0 (d, C-14), 50.2 (s, C-7), 63.2 (t, C-4), 72.2 (d, C-8), 88.9 (s, C-2), 117.7 (t, C-20), 128.4 (d, C-1), 148.0 (s, C-3), 151.4 (s, C-11), 176.8 (s, C-5), 205.4 (C-12); IR (film): ῡ_max_ 3425, 2969, 1758, 1726, 1647, 1461, 1246, 1199, 1020, 907 cm^−1^; EIMS *m*/*z* (relative intensity) 348 [M]^+^ (C_20_H_28_O_5_)^+^ (6), 333 [M-CH_3_]^+^ (46), 263 (100), 175 (21), 167 (59), 139 (23), 109 (25), 97 (39), 83 (21), 69 (50), 55 (36), 41 (60). Anal. Calcd for C_20_H_28_O_5_: C, 68.94; H, 8.10. Found: C, 69.15; H, 8.18.

#### 4.3.2. Tricholomalide D (**24**)

Colorless, sticky oil; [α]_D_^22^ + 31.2 (*c* 10.9 mg/mL, CH_2_Cl_2_); R_f_ 0.53 (silica gel TLC; CH_2_Cl_2_-Me_2_CO, 30:1); CD (MeOH) Δε_220_ −0.45, Δε_246_ +0.34, Δε_350_ +0.11; ^1^H and ^13^C NMR spectral data, see Table S1 in the Supplementary Materials; IR (film): ῡ_max_ 2968, 1783, 1736, 1699, 1648, 1367, 1241, 1027, 990, 961 cm^−1^; EIMS *m*/*z* (relative intensity) 388 [M]^+^ (C_22_H_28_O_6_)^+^ (54), 359 [M-CHO]^+^ (11), 346 [M-CH_2_CO]^+^ (18), 328 [M-AcOH]^+^ (34), 313 (12), 285 (45), 269 (21), 263 (33), 178 (23), 163 (48), 133 (37), 106 (78), 95 (33), 83 (42), 69 (35), 58 (69), 43 (100). Anal. Calcd for C_22_H_28_O_6_: C, 66.02; H, 7.27. Found: C, 66.15; H, 7.44.

#### 4.3.3. Tricholomalide E (**25**)

Colorless, sticky oil; [α]_D_^22^ + 1.2 (*c* 5.2 mg/mL, CH_2_Cl_2_); R_f_ 0.67 (RP18 TLC; MeOH-H_2_O, 80:20); CD (MeOH) Δε_225_ −2.28, Δε_246_ +0.11, Δε_272_ −0.68, Δε_350_ +0.46; ^1^H and ^13^C NMR spectral data, see Table S1 in the Supplementary Materials; IR (film): ῡ_max_ 3474, 2929, 1780, 1723, 1696, 1647, 1461, 1375, 1246, 1024, 962 cm^−1^; EIMS *m*/*z* (relative intensity) 346 [M]^+^ (C_20_H_26_O_5_)^+^ (31), 287 (40), 263 (58), 231 (22), 203 (22), 191 (36), 173 (25), 167 (82), 163 (51), 151 (37), 133 (39), 121 (30), 109 (32), 91 (40), 83 (61), 69 (68), 55 (68), 43 (100). Anal. Calcd for C_20_H_26_O_5_: C, 69.34; H, 7.57. Found: C, 69.75; H, 7.78.

#### 4.3.4. Tricholomalide F (**26**)

Colorless, sticky oil; [α]_D_^22^ + 63.9 (*c* 1.8 mg/mL, CH_2_Cl_2_); R_f_ 0.57 (RP18 TLC; MeOH-H_2_O, 80:20); CD (MeOH) Δε_255_ +0.47, Δε_275_ −0.05, Δε_340_ +0.20; ^1^H and ^13^C NMR spectral data, see Table S2 in the Supplementary Materials; IR (film): ῡ_max_ 3561, 3416, 2941, 2876, 1783, 1765, 1725, 1638, 1461, 1371, 1242, 1051, 976 cm^−1^; EIMS *m*/*z* (relative intensity) 346 [M]^+^ (C_20_H_26_O_5_)^+^ (30), 331 [M-CH_3_]^+^ (9), 317 [M-CHO]^+^ (33), 303 (35), 287 (23), 263 (99), 235 (46), 203 (29), 191 (61), 175 (27), 163 (61), 149 (43), 135 (48), 121 (33), 109 (38), 97 (47), 83 (58), 69 (84), 55 (88), 43 (70), 41 (100). Anal. Calcd for C_20_H_26_O_5_: C, 69.34; H, 7.57. Found: C, 69.65; H, 7.73.

#### 4.3.5. Tricholomalide G (**27**)

Colorless, sticky oil; [α]_D_^22^ + 55.2 (*c* 4.8 mg/mL, CH_2_Cl_2_); R_f_ 0.57 (RP18 TLC; MeOH-H_2_O, 80:20); CD (MeOH) Δε_250_ +0.43, Δε_345_ +0.18; ^1^H and ^13^C NMR spectral data, see Table S1 in the Supplementary Materials; IR (film): ῡ_max_ 3449, 3060, 2960, 2929, 1772, 1731, 1644, 1463, 1370, 1241, 1020, 963, 938, 735 cm^−1^; EIMS *m*/*z* (relative intensity) 390 [M]^+^ (C_22_H_30_O_6_)^+^ (9), 375 [M-CH_3_]^+^ (54), 315 [M-AcOH]^+^ (15), 263 (45), 191 (17), 181 (24), 167 (32), 135 (15), 109 (16), 95 (24), 69 (39), 55 (27), 43 (100), 41 (44). Anal. Calcd for C_22_H_30_O_6_: C, 67.67; H, 7.74. Found: C, 67.81; H, 7.62.

### 4.4. Acetylation of Tricholomalide E (**25**) to Tricholomalide D (**24**)

Tricholomalide E (**25**) (3 mg, 8.7 μmol) in CH_2_Cl_2_ (1 mL) was added to pyridine (50 μL), followed by excess Ac_2_O (50 μL). The mixture was stirred for 2 h at 22 °C, quenched with MeOH (0.5 mL), and evaporated. The residue was filtered through a short pad of silica gel (300 mg). Elution with CH_2_Cl_2_-MeOH at 99:1 gave a product (3.3 mg, 99%) identical ([α]_D_^22^, NMR data) to tricholomalide D (**24**).

### 4.5. Oxidation of Tricholomalide G (**27**) to Tricholomalide D (**24**) and Tricholomalide C (**17**) to Tricholomalide E (**25**)

Freshly prepared PDC (9 mg, 24 μmol) [26] was added in one portion to tricholomalide G (**27**) (3 mg, 7.7 μmol) dissolved in CH_2_Cl_2_ (0.5 mL). The mixture was stirred for 6 h at 22 °C; subsequently, it was diluted with abundant Et_2_O. The organic layer was washed with aqueous NaHCO_3_, dried (Na_2_SO_4_), and evaporated. The residue was filtered through a short pad of silica gel (300 mg). Elution with CH_2_Cl_2_-MeOH at 99:1 gave a product (2.3 mg, 77%) identical ([α]_D_^22^, NMR data) to tricholomalide D (**24**).

Isolated tricholomalide C (**17**) was converted to tricholomalide E (**25**) in 75% yield using the procedure described above.

## 5. Conclusions

The isolation of four novel seconeodolastane diterpenoids, tricholomalides D–G, from *T. ustaloides* has confirmed that the fruiting bodies of *Tricholoma* mushrooms are rich sources of new compounds, most of which have unique chemical structures. The rare seconeodolastane skeleton of tricholomalides seems to be a characteristic feature of the diterpenoids present in the fruiting bodies of *Tricholoma*; however, epimeric derivatives, e.g., compounds **7** and **24**, are produced by different species, and this finding may be chemotaxonomically significant. So far, besides being isolated from fruiting bodies of the genus *Tricholoma*, seconeodolastane diterpenoids have only been isolated from a mycelial culture of *Lepista sordida* [27]. Interestingly, the genera *Tricholoma* and *Lepista* both belong to the family *Tricholomataceae* (*Agaricales*).

In the future, we will extend our investigations to the chemical contents of other *Tricholoma* species grown in the Italian woods, and, regarding tricholomalides C–G, we intend to study their biological activities using in vitro tests.

## Data Availability

Raw data are available from one of the authors (G.V.).

## Supplementary Materials

The following supporting information can be downloaded at https://www.mdpi.com/article/10.3390/molecules28217446/s1: ^1^H NMR graphical spectra of tricholomalides C–G; COSY graphical spectra of tricholomalides D–G; ^13^C NMR graphical spectra of tricholomalides C–G; DEPT ^13^C NMR graphical spectra of tricholomalides C–G; EIMS graphical spectra of tricholomalides D–G; CD graphical spectra of tricholomalides C–G; tabulated NMR spectral data of tricholomalides D–G.
