# Supplementary material for: New Tricholomalides D–G from the Mushroom Tricholoma ustaloides Grown in an Italian Beech Wood
Source: Molecules. 2023 Nov 6;28(21):7446. doi: 10.3390/molecules28217446 (PMC10650213; doi:10.3390/molecules28217446)
Supplement: Supplementary file 1 [file molecules-28-07446-s001.zip › molecules-2653564-supplementary.pdf]

SUPPLEMENTARY INFORMATION

New Tricholomalides D-G from the Mushroom *Tricholoma ustaloides* Grown in an Italian Beech Wood

Gianluca Gilardoni [1], Francesca Negri [2], Paola Vita Finzi [2], Faiq H. S. Hussain [3], and Giovanni Vidari [2,3,*]

[1] Departamento de Química, Universidad Técnica Particular de Loja (UTPL), Loja 110107, Ecuador; ggilardoni@utpl.edu.ec or gianluca.gilardoni@gmail.com (G.G.)
[2] Dipartimento di Chimica, Università degli Studi di Pavia, Via Taramelli 10, 27100 Pavia, Italy; f.negri@mail.com (F.N.); paola.vitafinzi@unipv.it (P.V.F.)
[3] Department of Medical Analysis, Faculty of Applied Science, Tishk International University, Erbil 44001, Iraq; faiq.hussain@tiu.edu.iq (F.H.S.H.)
* Correspondence: vidari@unipv.it (G.V.)

page

S1. ^{1}H NMR spectrum of Tricholomalide C (**17**). 3
S2. ^{13}C NMR spectrum of Tricholomalide C (**17**). 4
S3. DEPT ^{13}C NMR spectrum of Tricholomalide C (**17**). 5
S4. CD spectrum of Tricholomalide C (**17**). 6
S5. ^{1}H NMR spectrum of Tricholomalide D (**24**). 7
S6. COSY spectrum of Tricholomalide D (**24**). 8
S7. ^{13}C NMR spectrum of Tricholomalide D (**24**). 9
S8. DEPT ^{13}C NMR spectrum of Tricholomalide D (**24**). 10
S9. EIMS spectrum of Tricholomalide D (**24**). 11
S10. CD spectrum of Tricholomalide D (**24**). 12
S11. ^{1}H NMR spectrum of Tricholomalide E (**25**). 13
S12. COSY spectrum of Tricholomalide E (**25**). 14
S13. ^{13}C NMR spectrum of Tricholomalide E (**25**). 15
S14. DEPT ^{13}C NMR spectrum of Tricholomalide E (**25**). 16

S15. EIMS spectrum of Tricholomalide E (**27**). 17
S16. CD spectrum of Tricholomalide E (**27**). 18
S17. ^{1}H NMR spectrum of Tricholomalide F (**28**). 19
S18. COSY spectrum of Tricholomalide F (**28**). 20
S19. ^{13}C NMR spectrum of Tricholomalide F (**28**). 21
S20. DEPT ^{13}C NMR spectrum of Tricholomalide F (**28**). 22
S21. EIMS spectrum of Tricholomalide F (**28**). 23
S22. CD spectrum of Tricholomalide F (**28**). 24
S23. ^{1}H NMR spectrum of Tricholomalide G (**29**). 25
S24. COSY spectrum of Tricholomalide G (**29**). 26
S25. ^{13}C NMR spectrum of Tricholomalide G (**29**). 27
S26. DEPT ^{13}C NMR spectrum of Tricholomalide G (**29**). 28
S27. EIMS spectrum of Tricholomalide G (**29**). 29
S28. CD spectrum of Tricholomalide G (**29**). 30
S29 Table 1. NMR spectral data for compounds **26**, **27**, and **29** in $CDCl_3$**.** 31
S30. Table 2. NMR spectral data for compound **28** in $CDCl_3$**.** 32

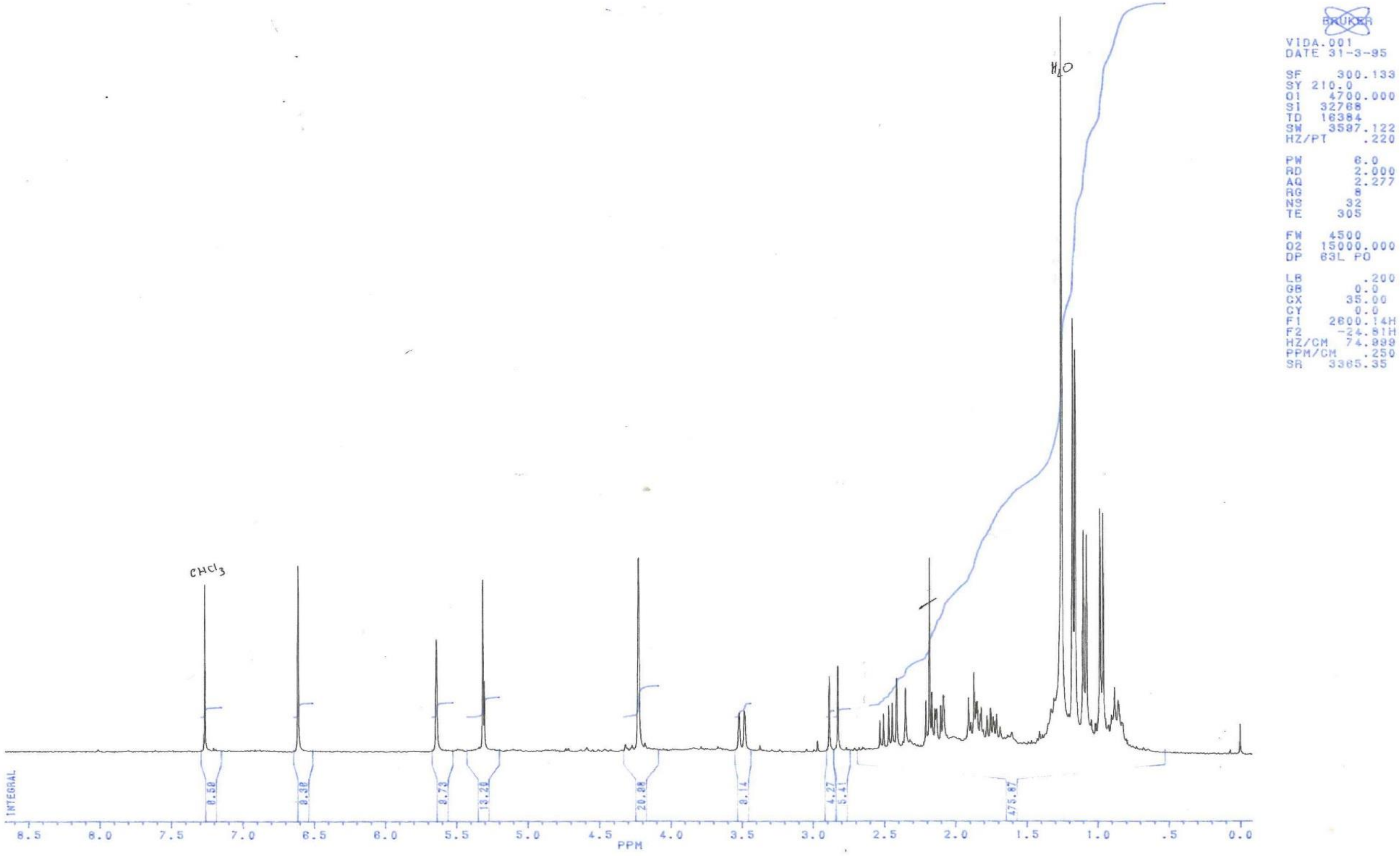

S1. ^{1}H NMR spectrum of Tricholomalide C (19).

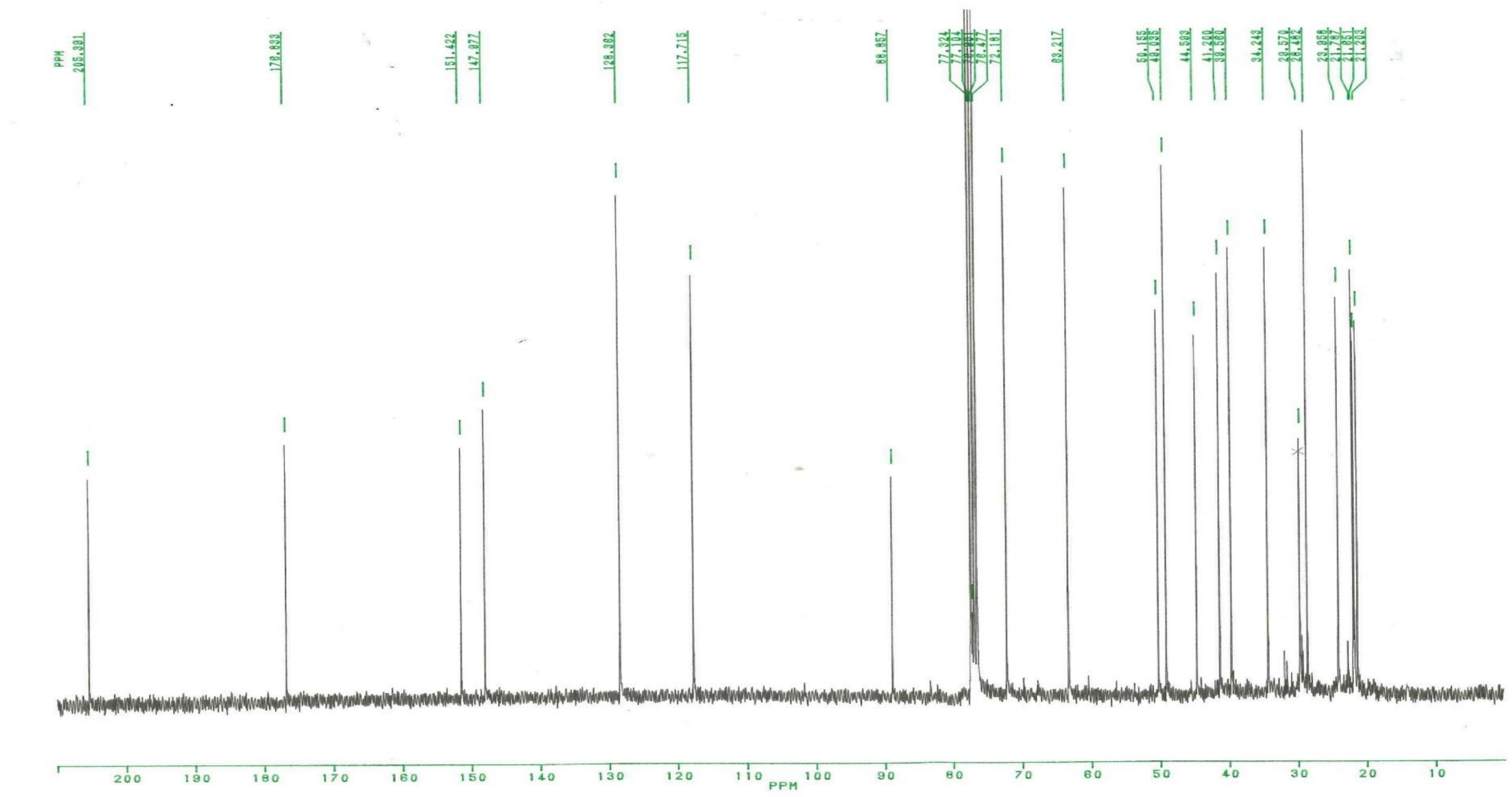

S2. ^{13}C NMR spectrum of Tricholomalide C (19).

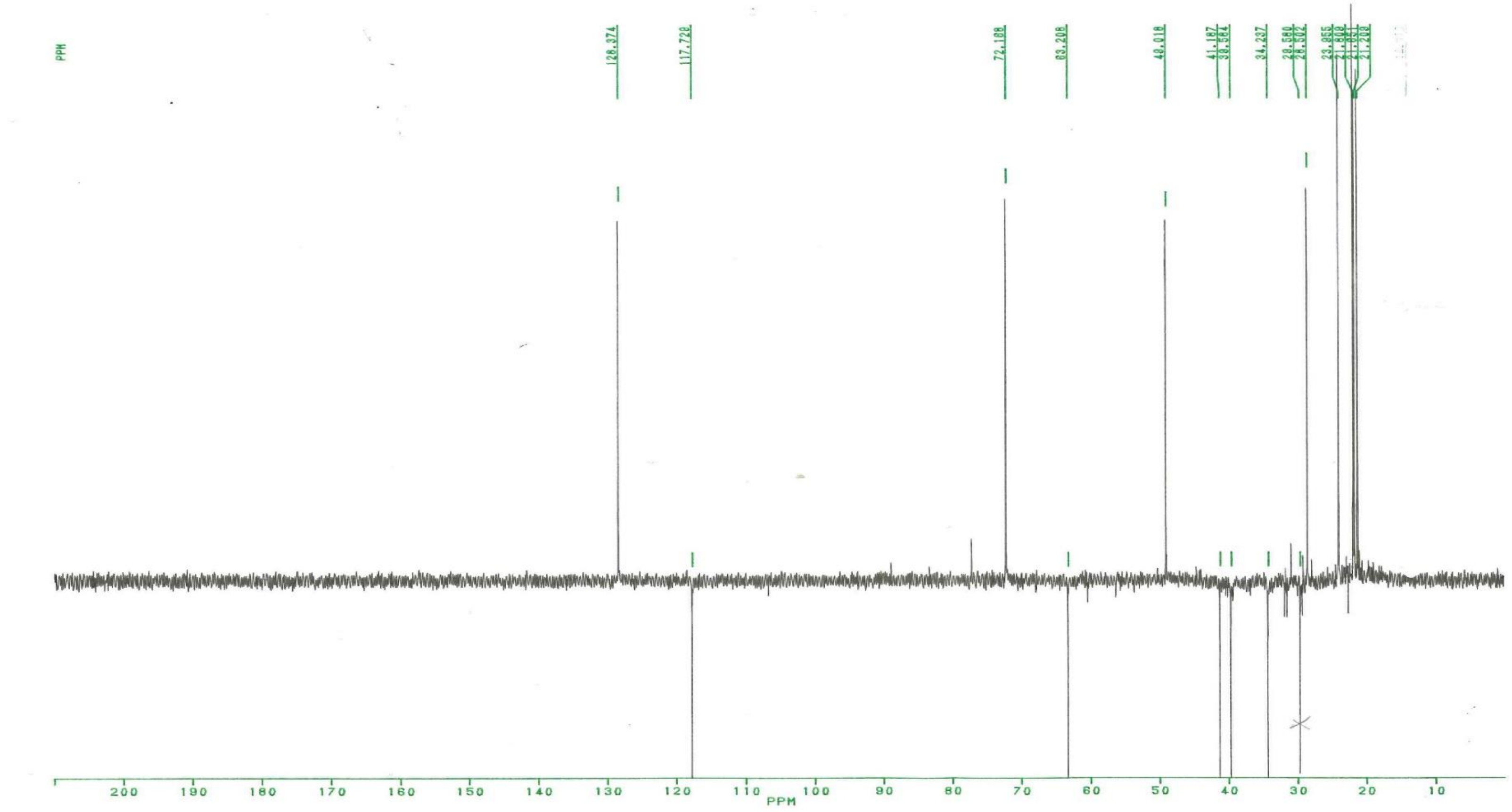

S3. DEPT ^{13}C NMR spectrum of Tricholomalide C (19).

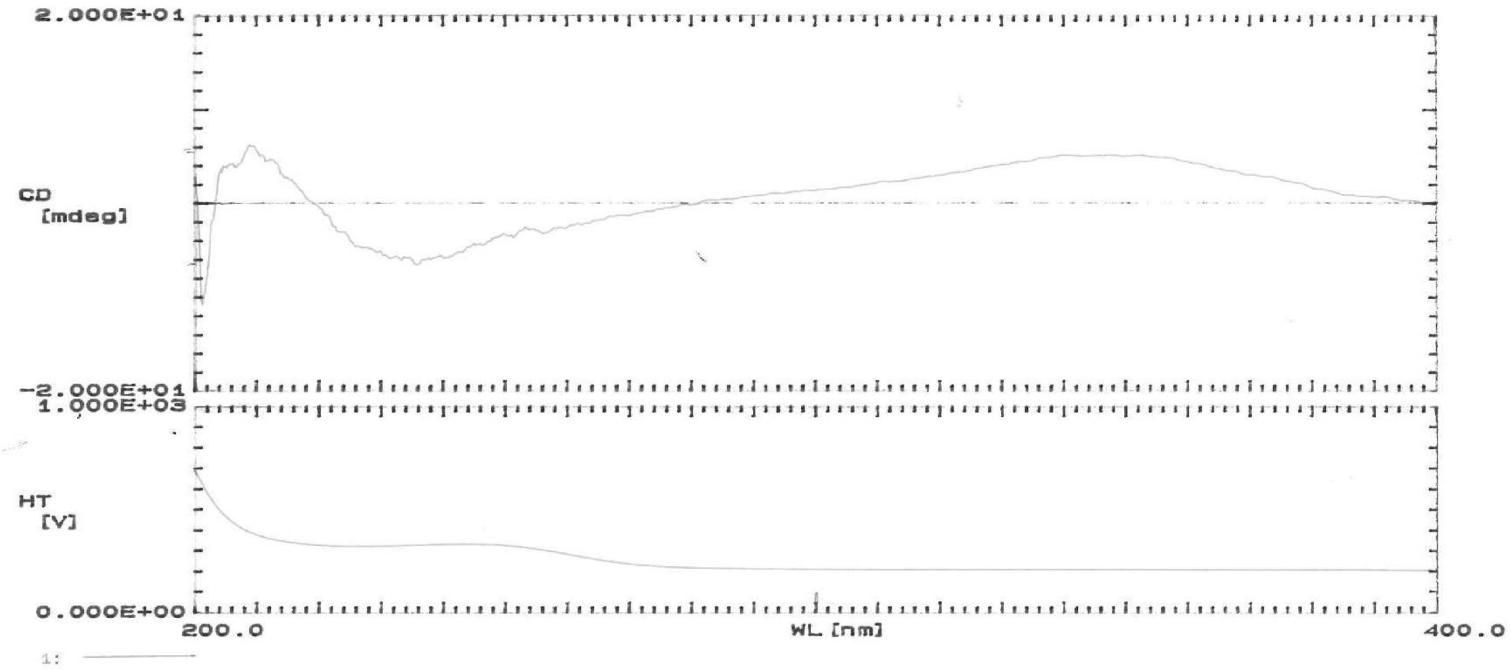

S4. CD spectrum of Tricholomalide C (19).

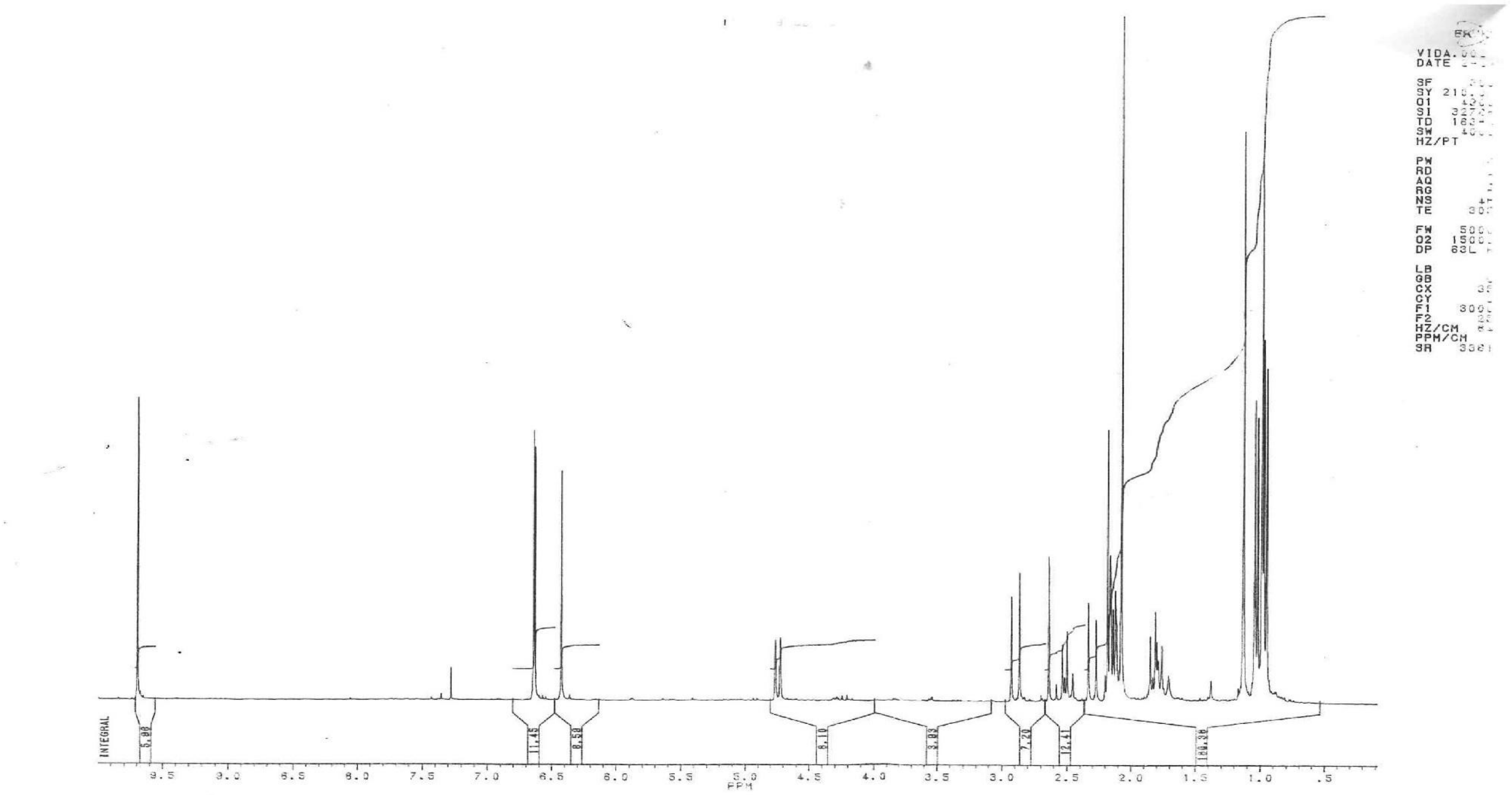

S5. ^{1}H NMR spectrum of Tricholomalide D (26).

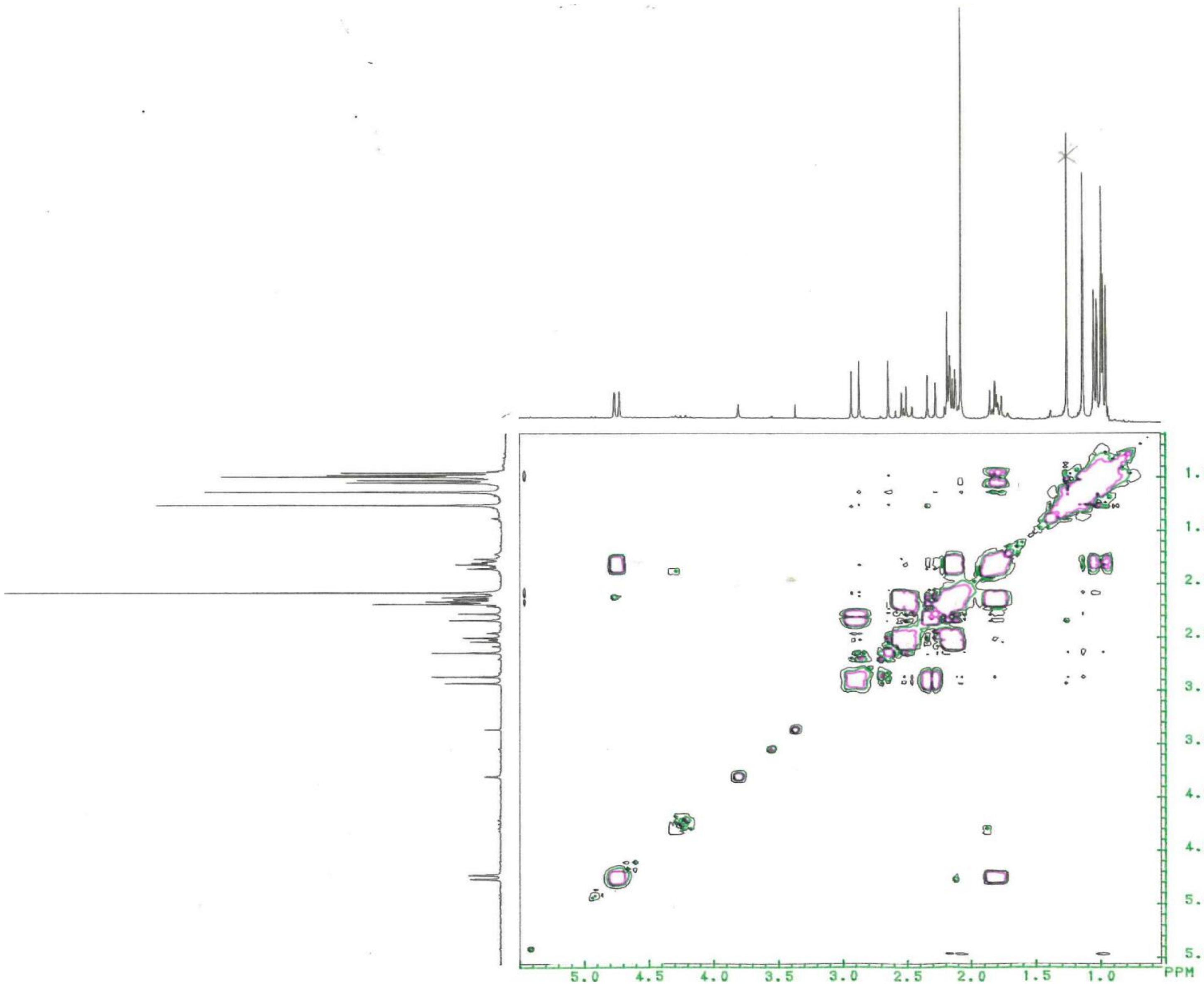

S6. COSY spectrum of Tricholomalide D (26).

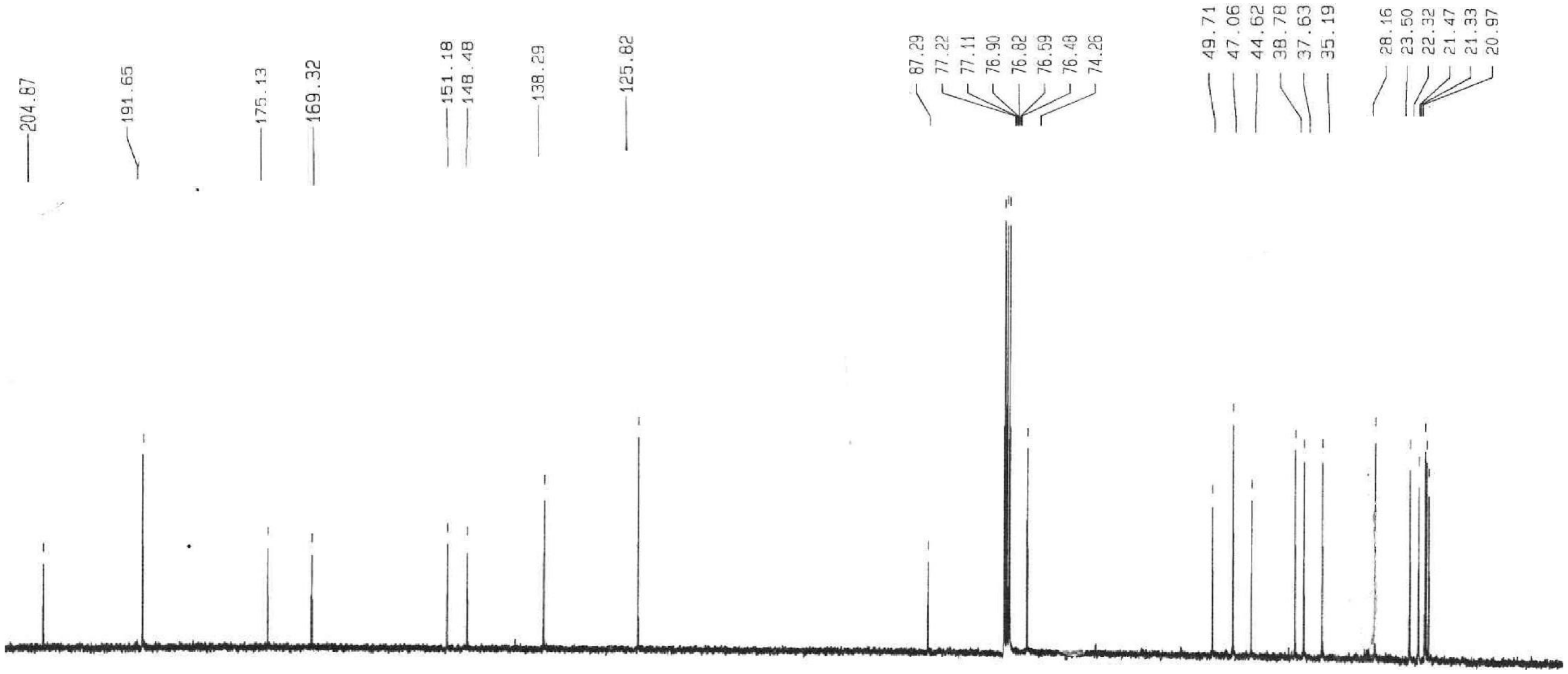

S7. ^{13}C spectrum of Tricholomalide D (26).

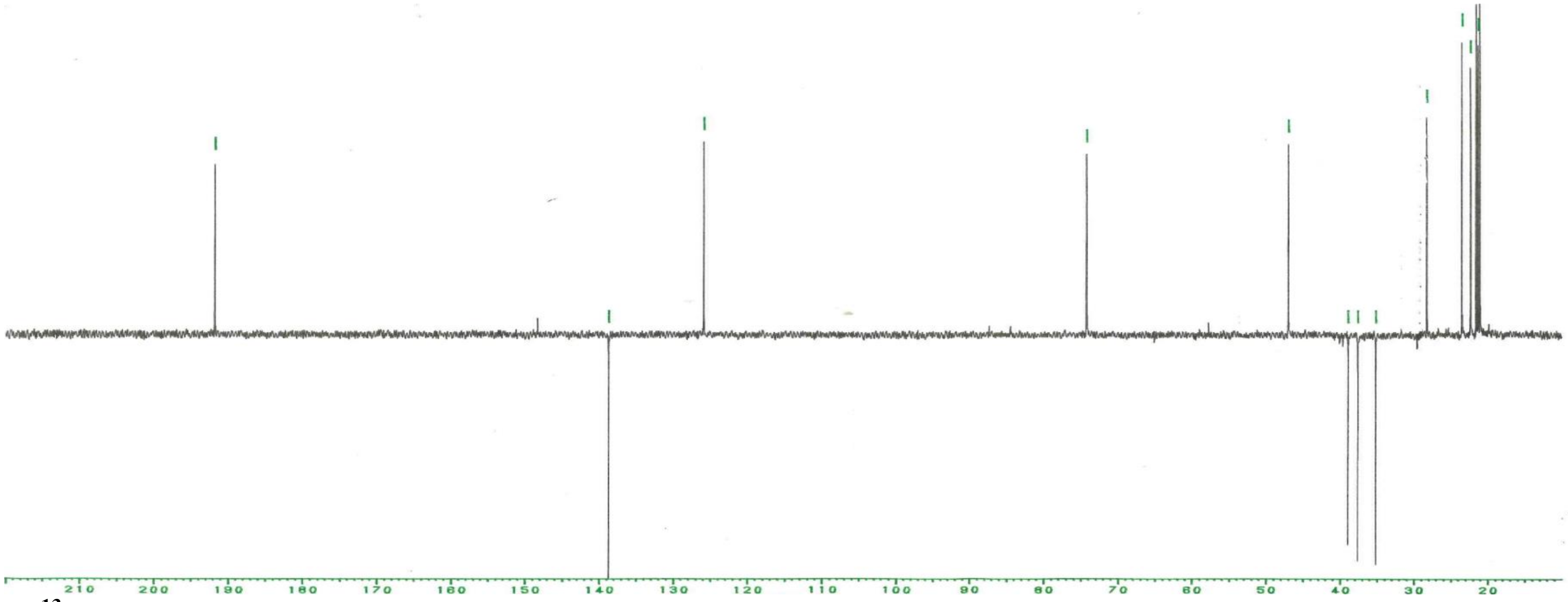

S8. DEPT ^{13}C spectrum of Tricholomalide D (26).

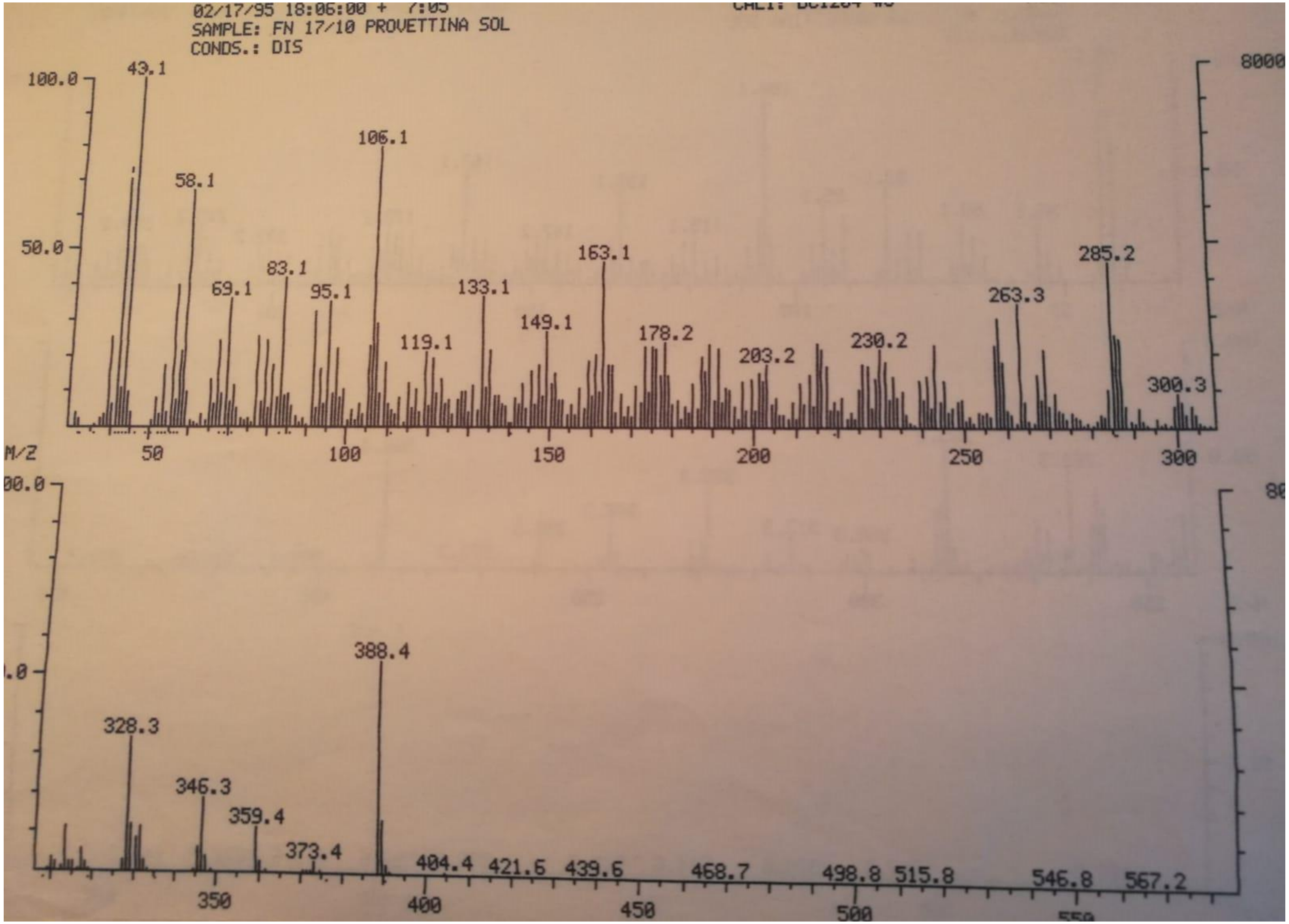

S9. EIMS spectrum of Tricholomalide D (26).

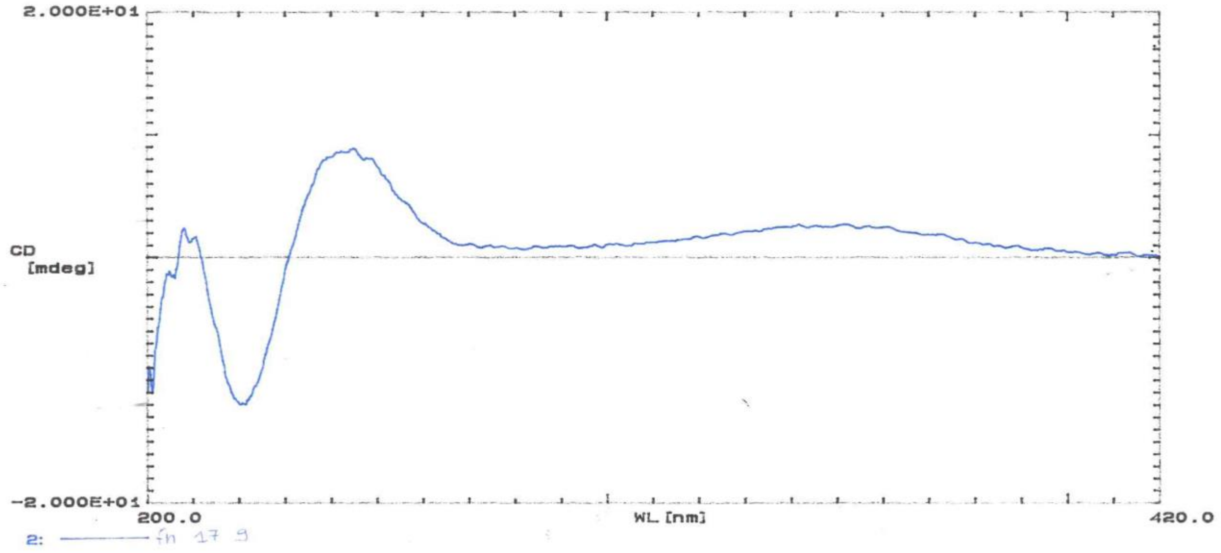

S10. CD spectrum of Tricholomalide D (26).

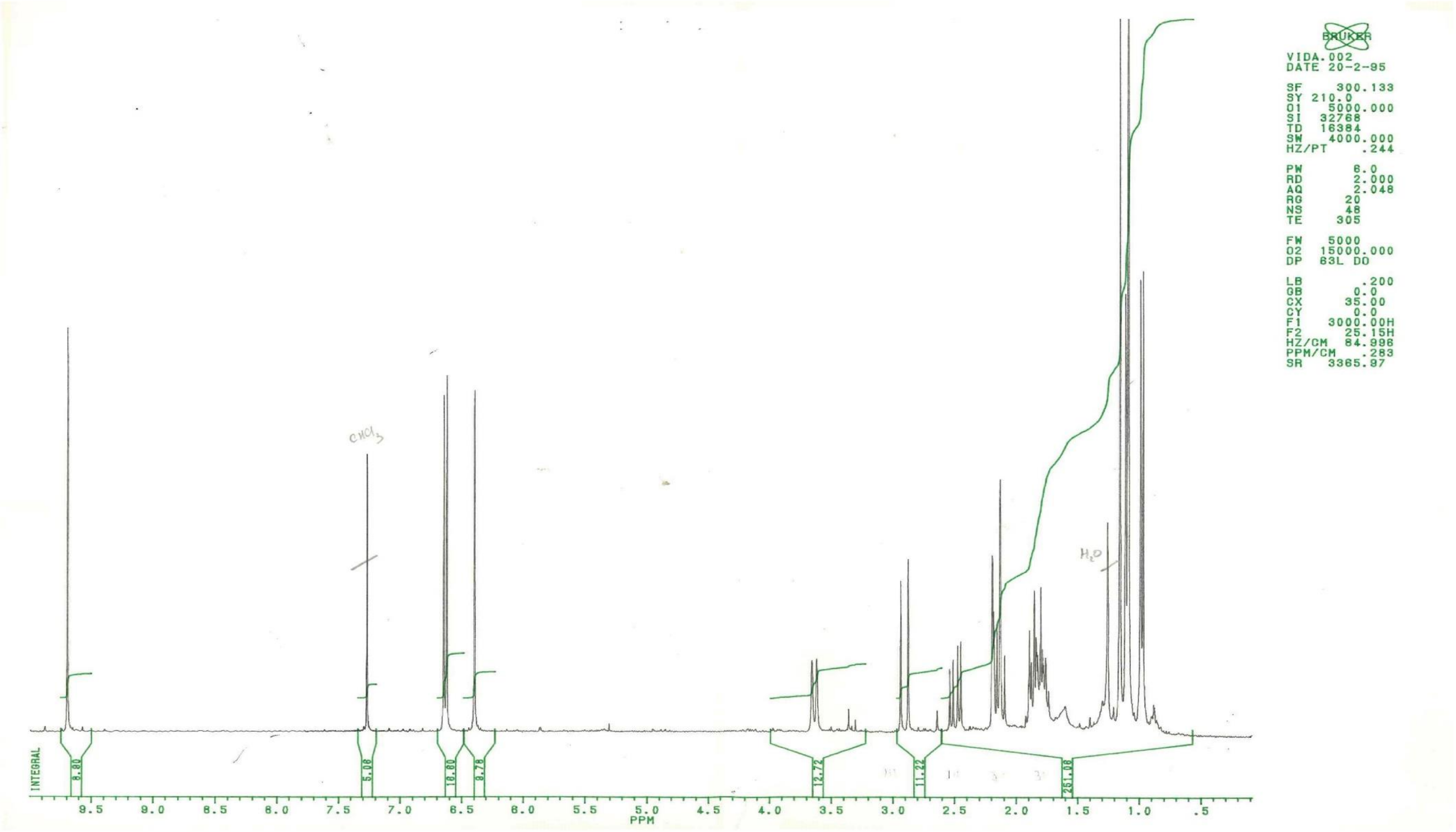

S11. ^{1}H NMR spectrum of Tricholomalide E (27).

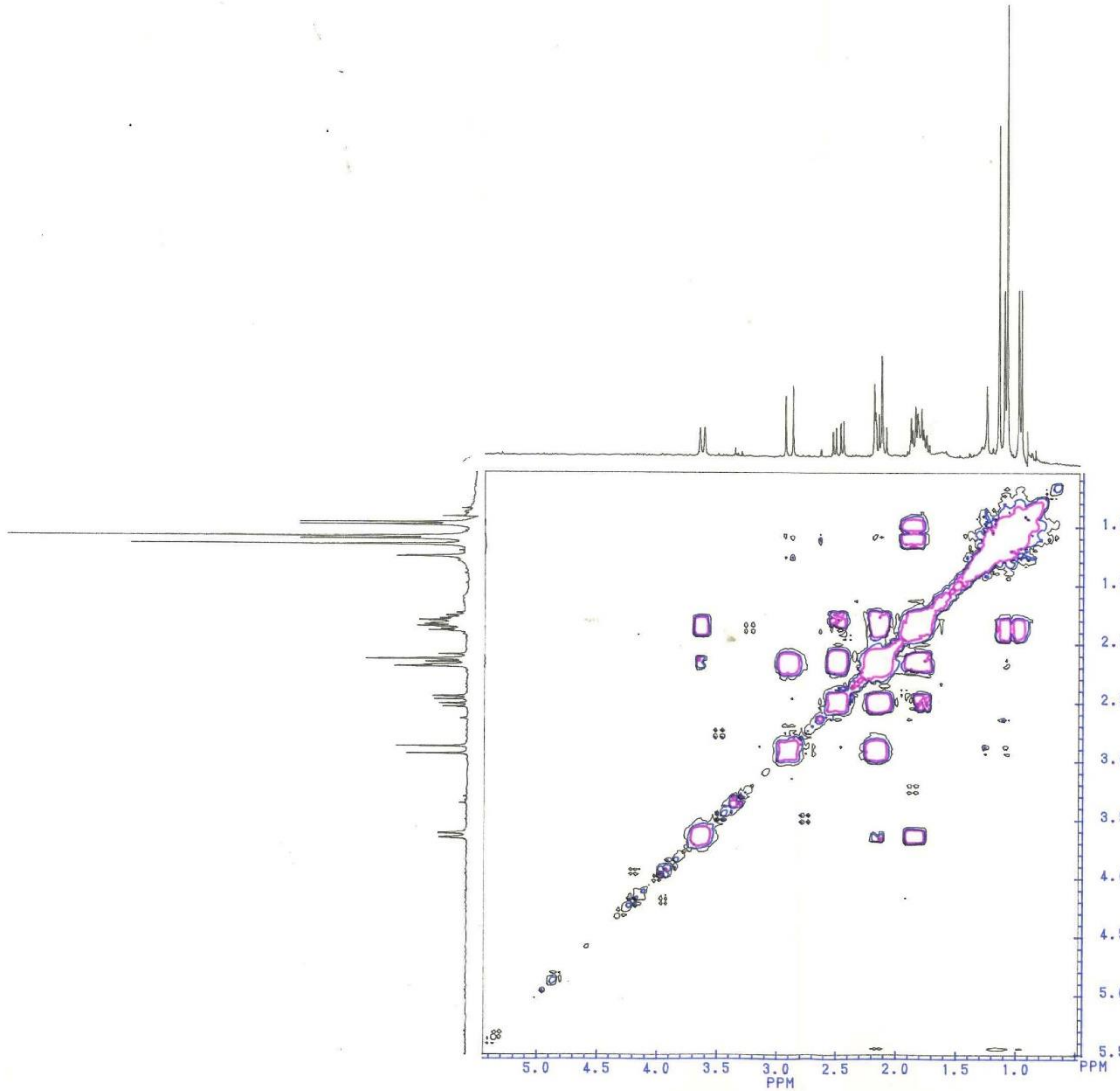

S12. COSY spectrum of Tricholomalide E (27).

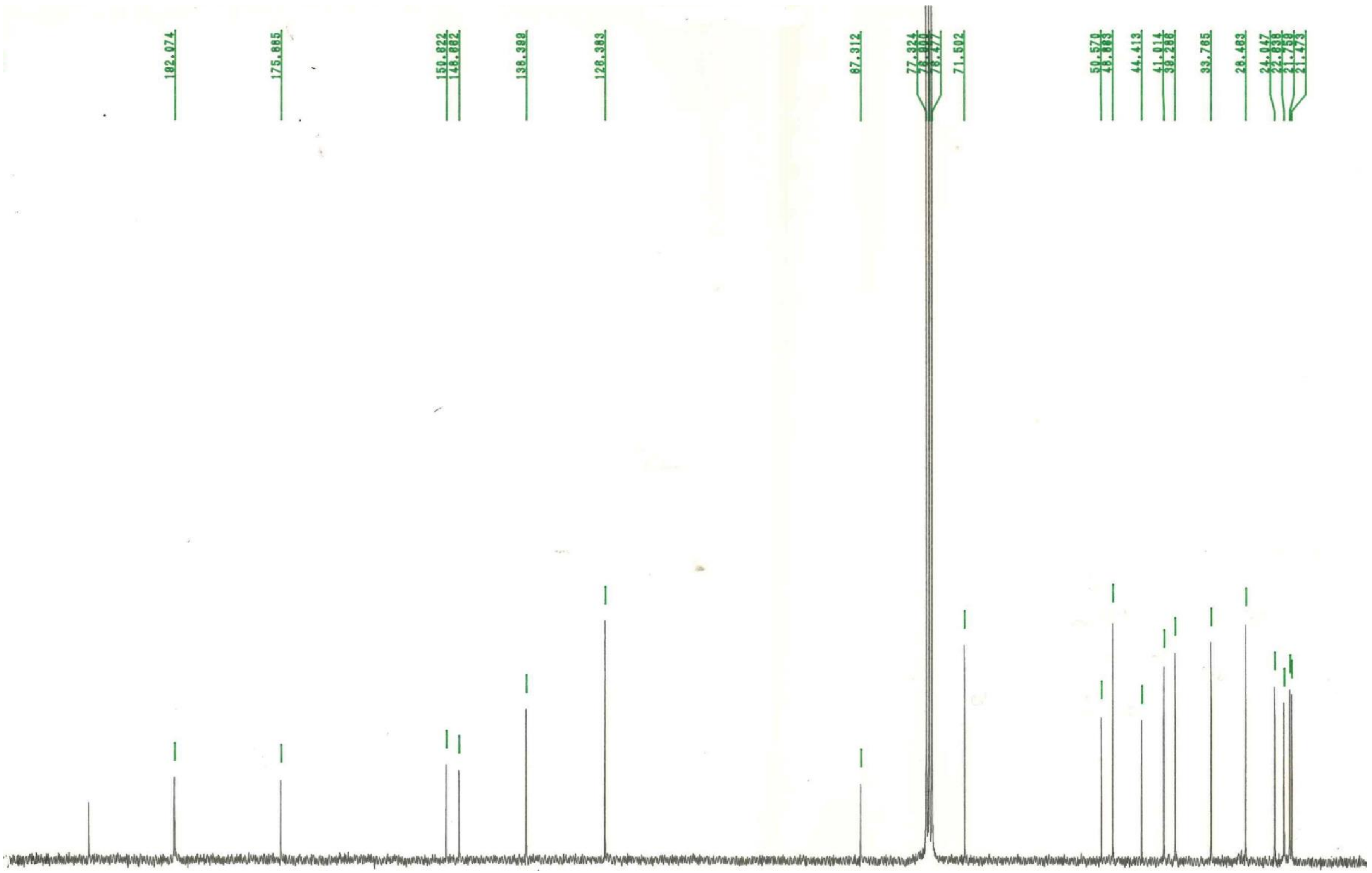

S13. ^{13}C NMR spectrum of Tricholomalide E (27).

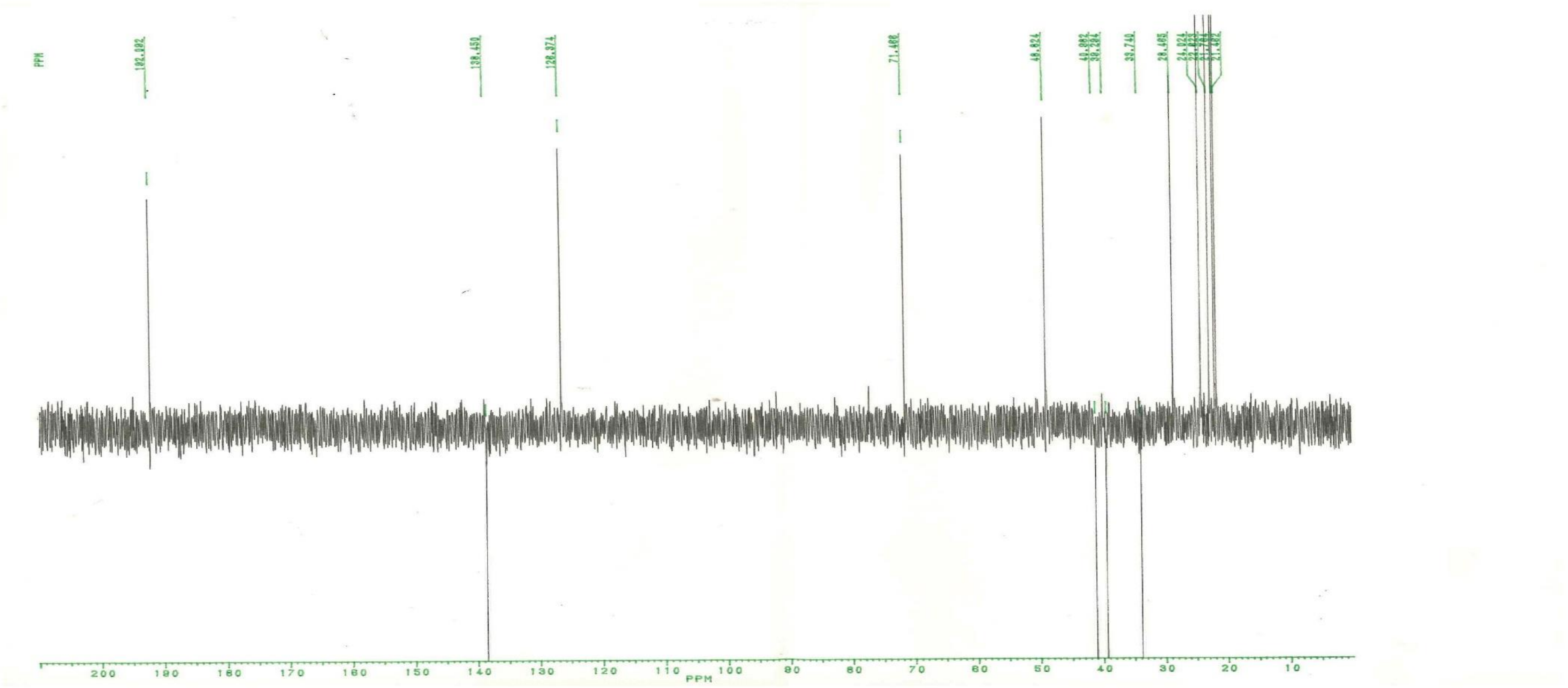

S14. DEPT ^{13}C NMR spectrum of Tricholomalide E (27).

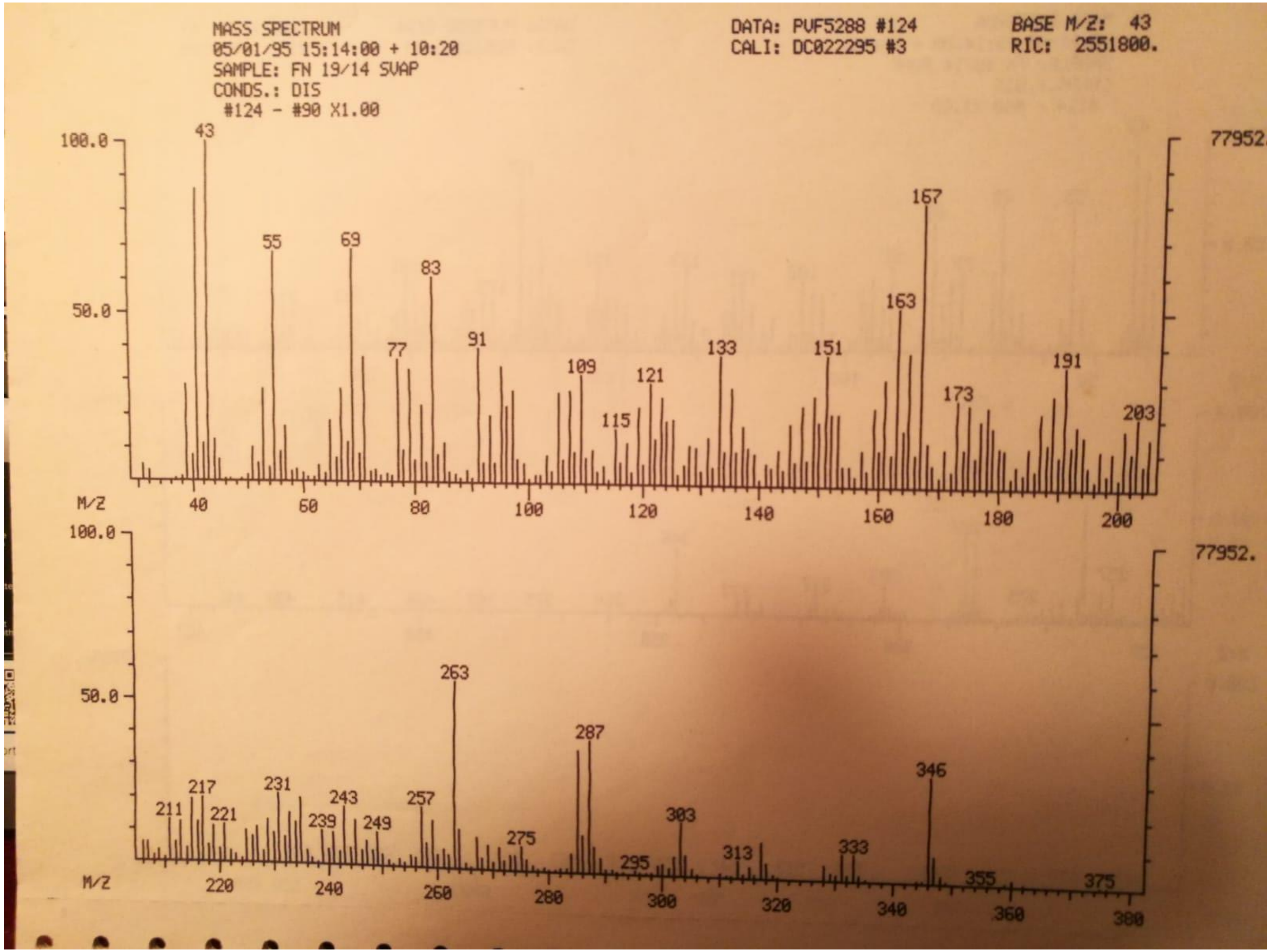

S15. EIMS spectrum of Tricholomalide E (27).

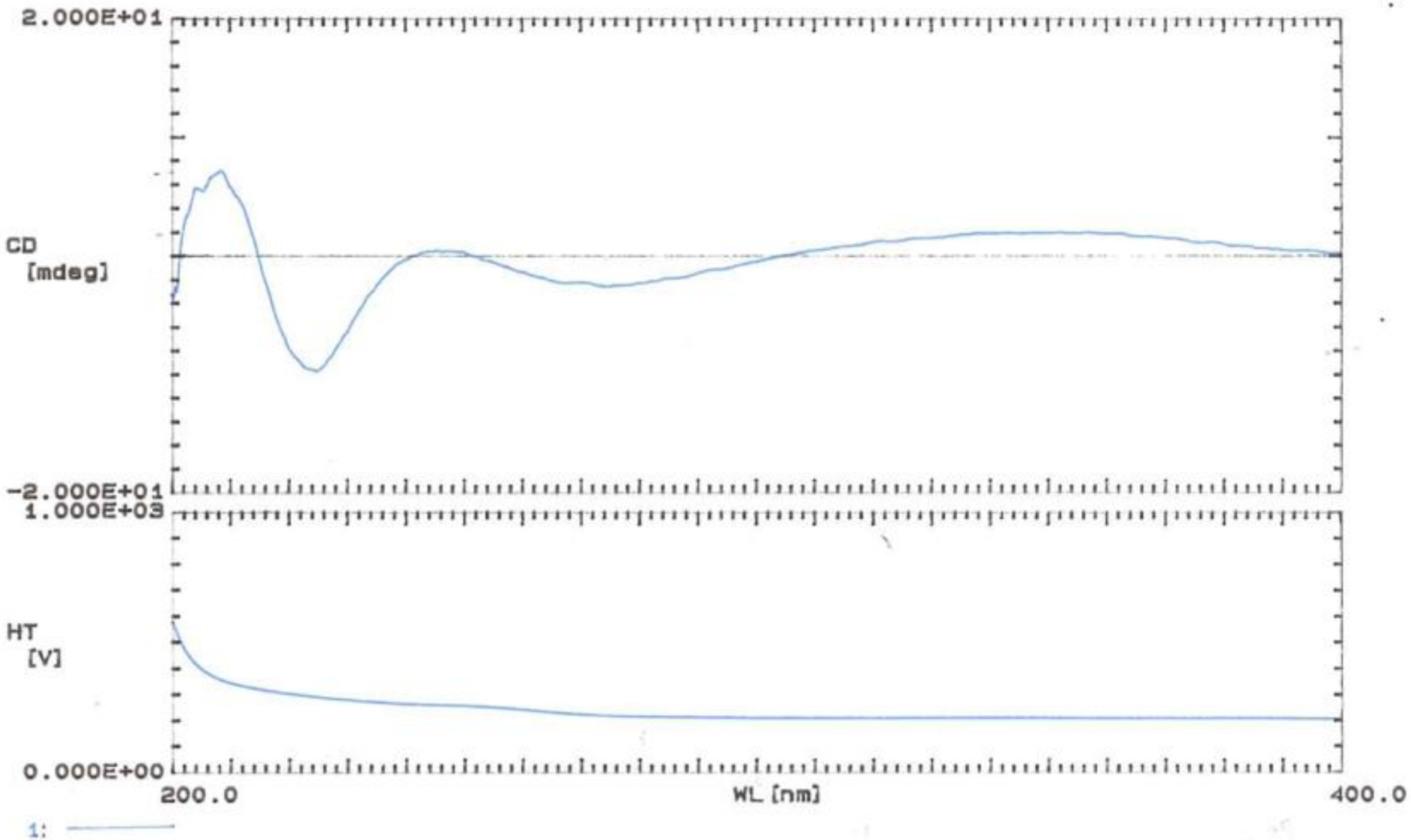

S16. CD spectrum of Tricholomalide E (27).

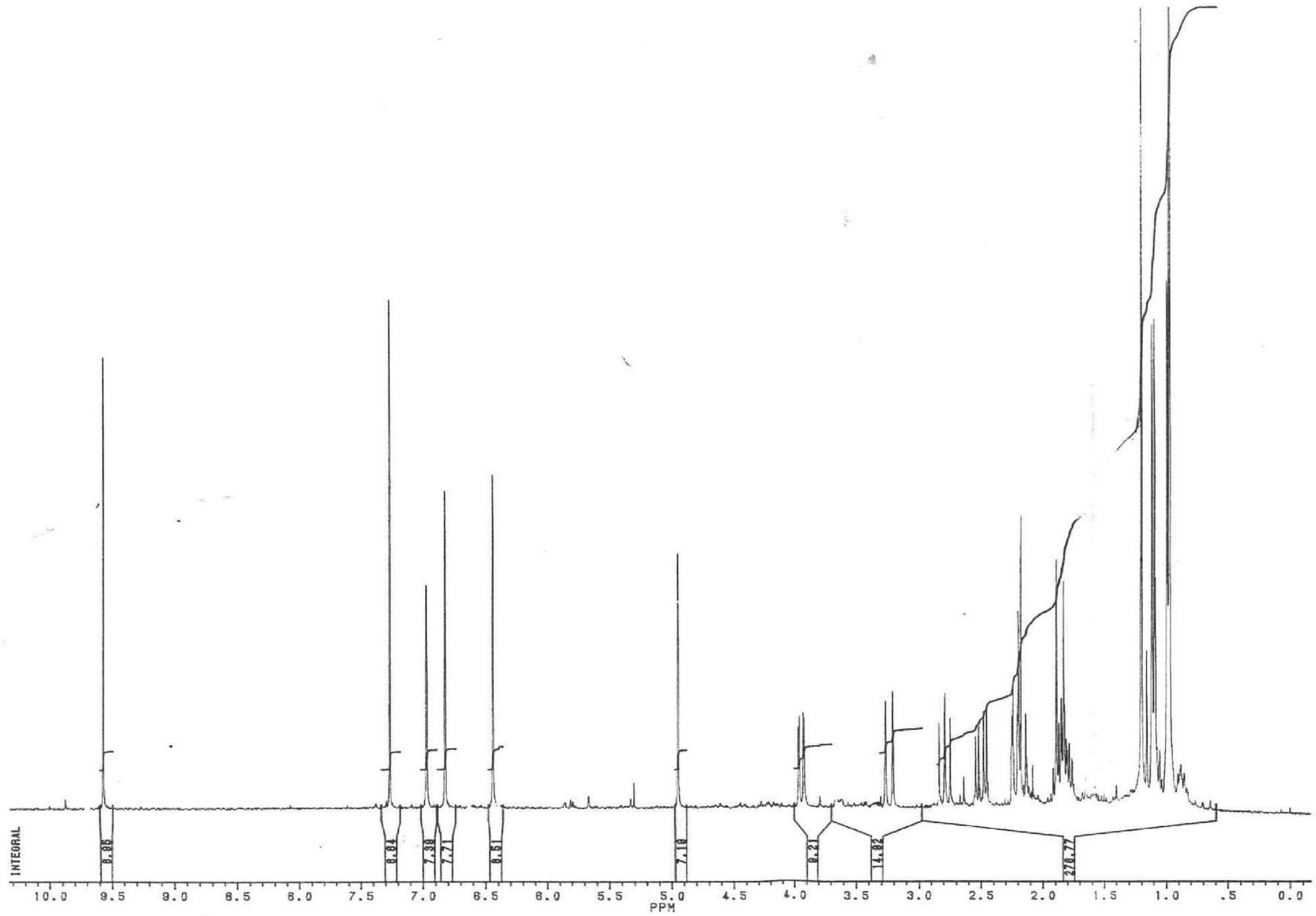

S17. ^{1}H NMR spectrum of Tricholomalide F (28).

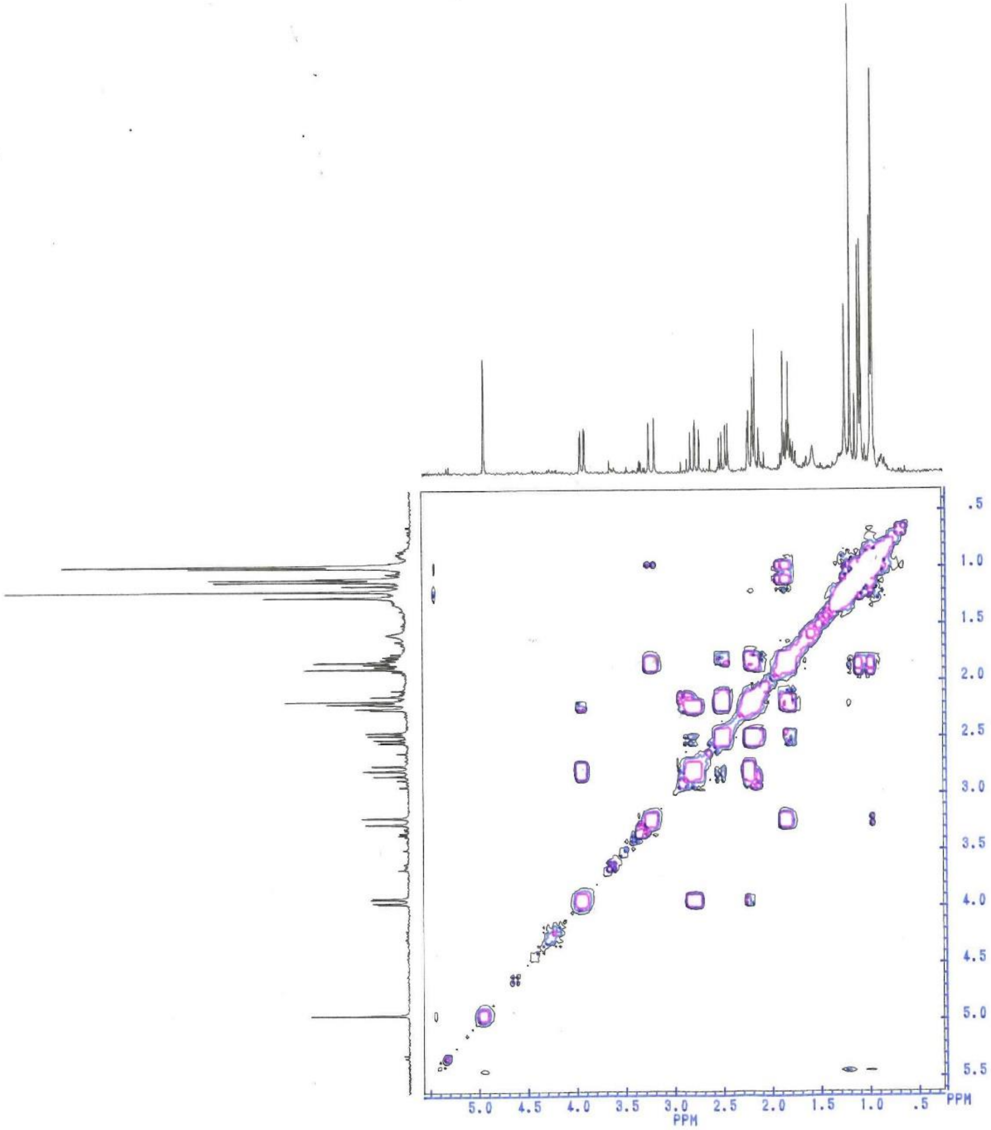

S18. COSY spectrum of Tricholomalide F (28).

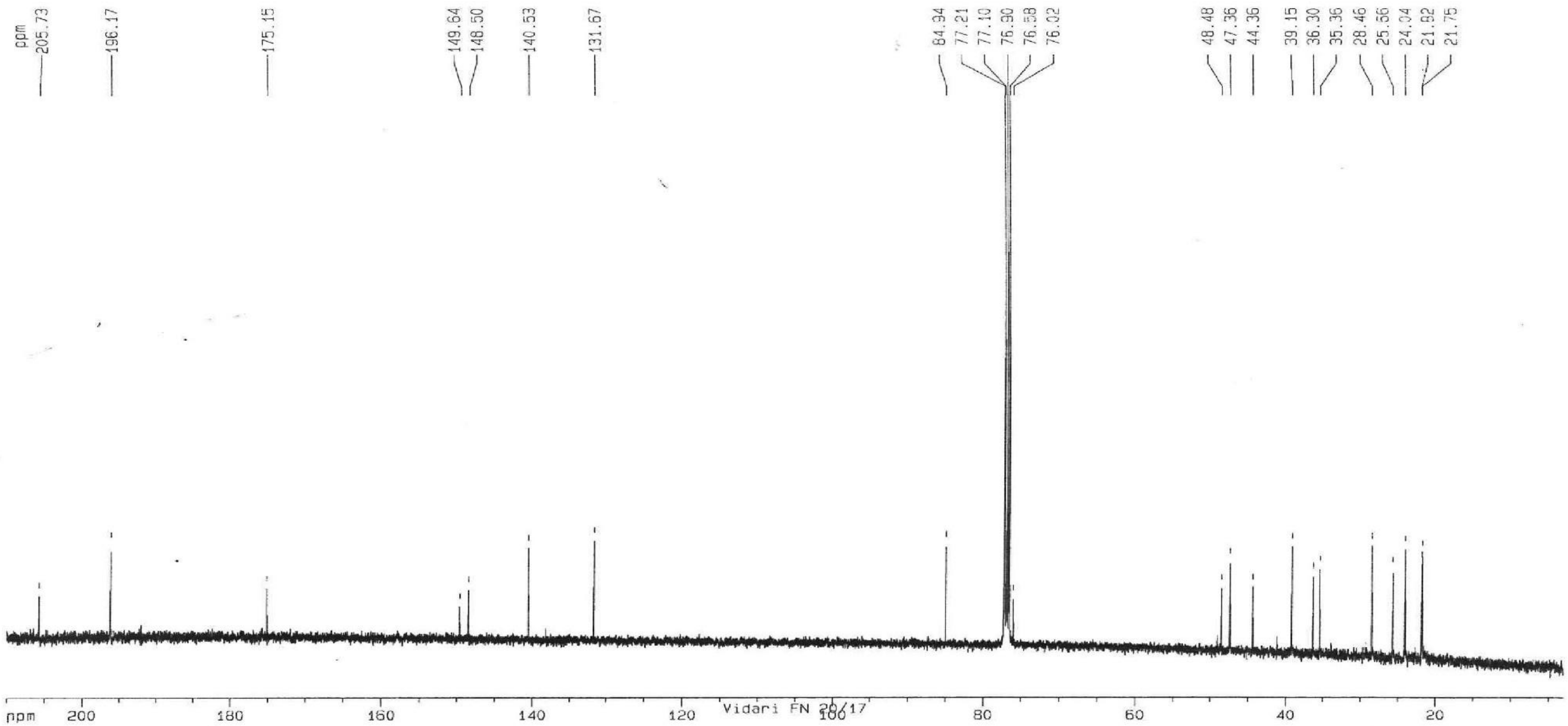

S19. ^{13}C NMR spectrum of Tricholomalide F (28).

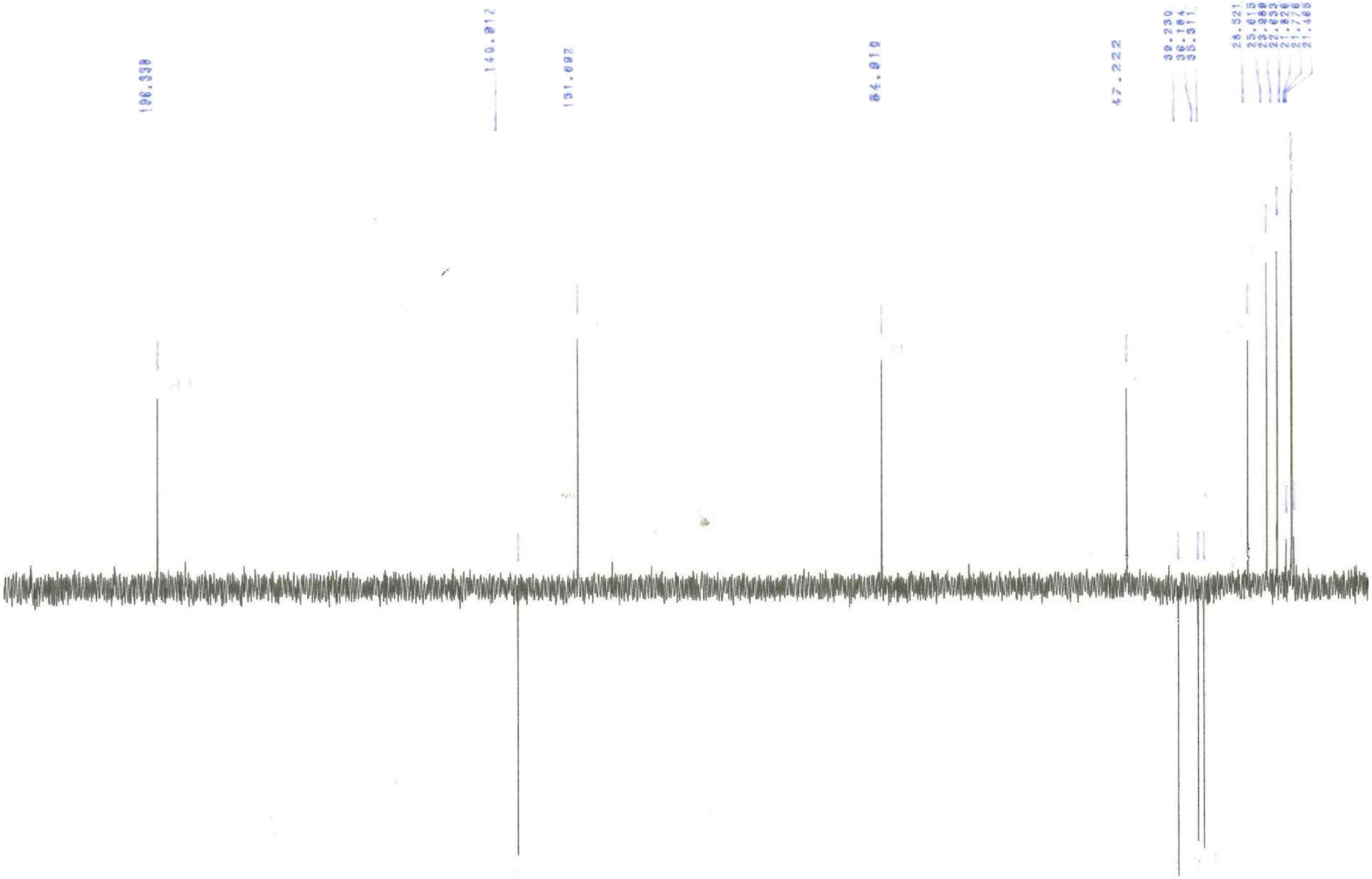

S20. DEPT ^{13}C NMR spectrum of Tricholomalide F (28).

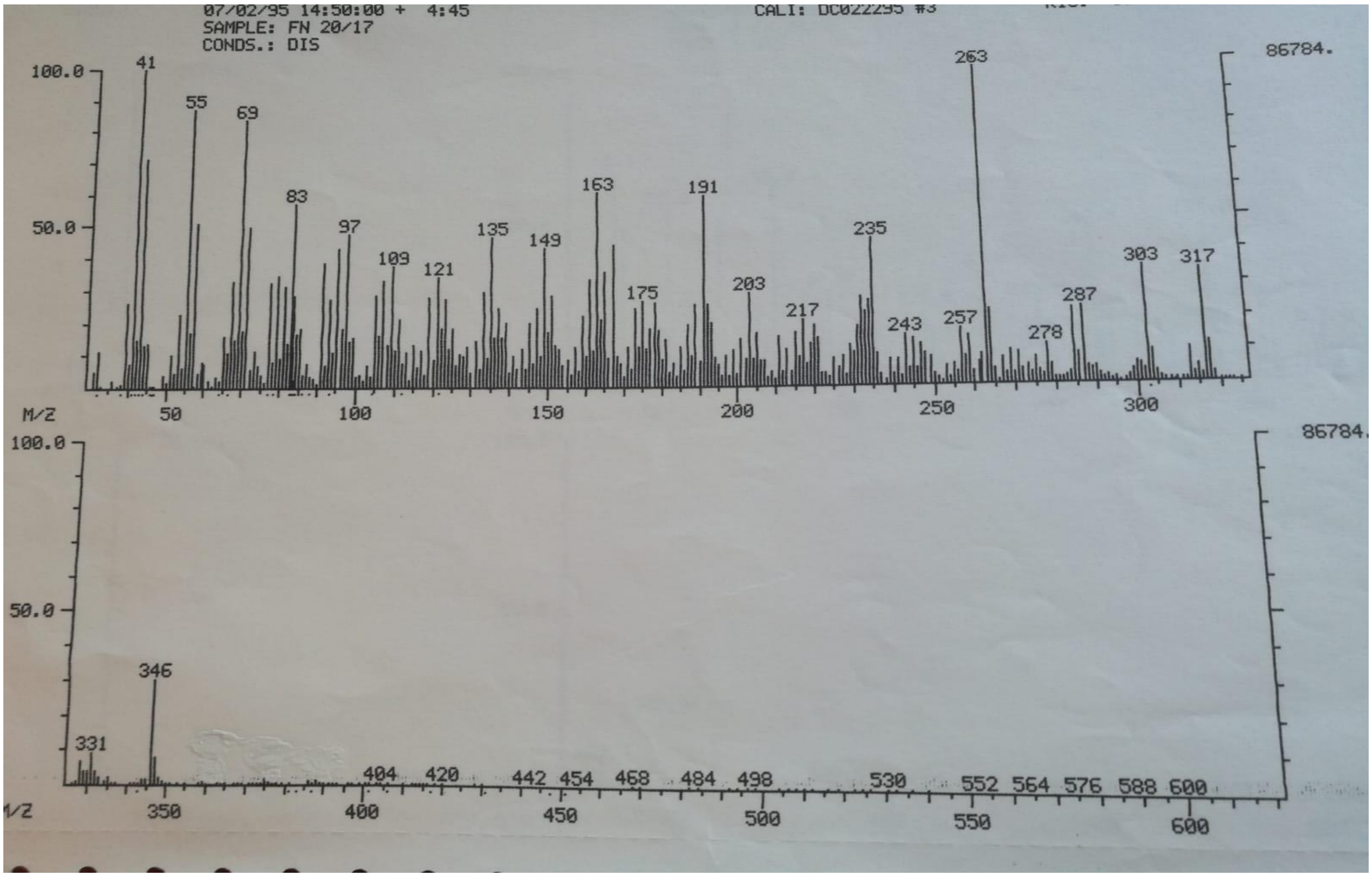

S21. EIMS spectrum of Tricholomalide F (28).

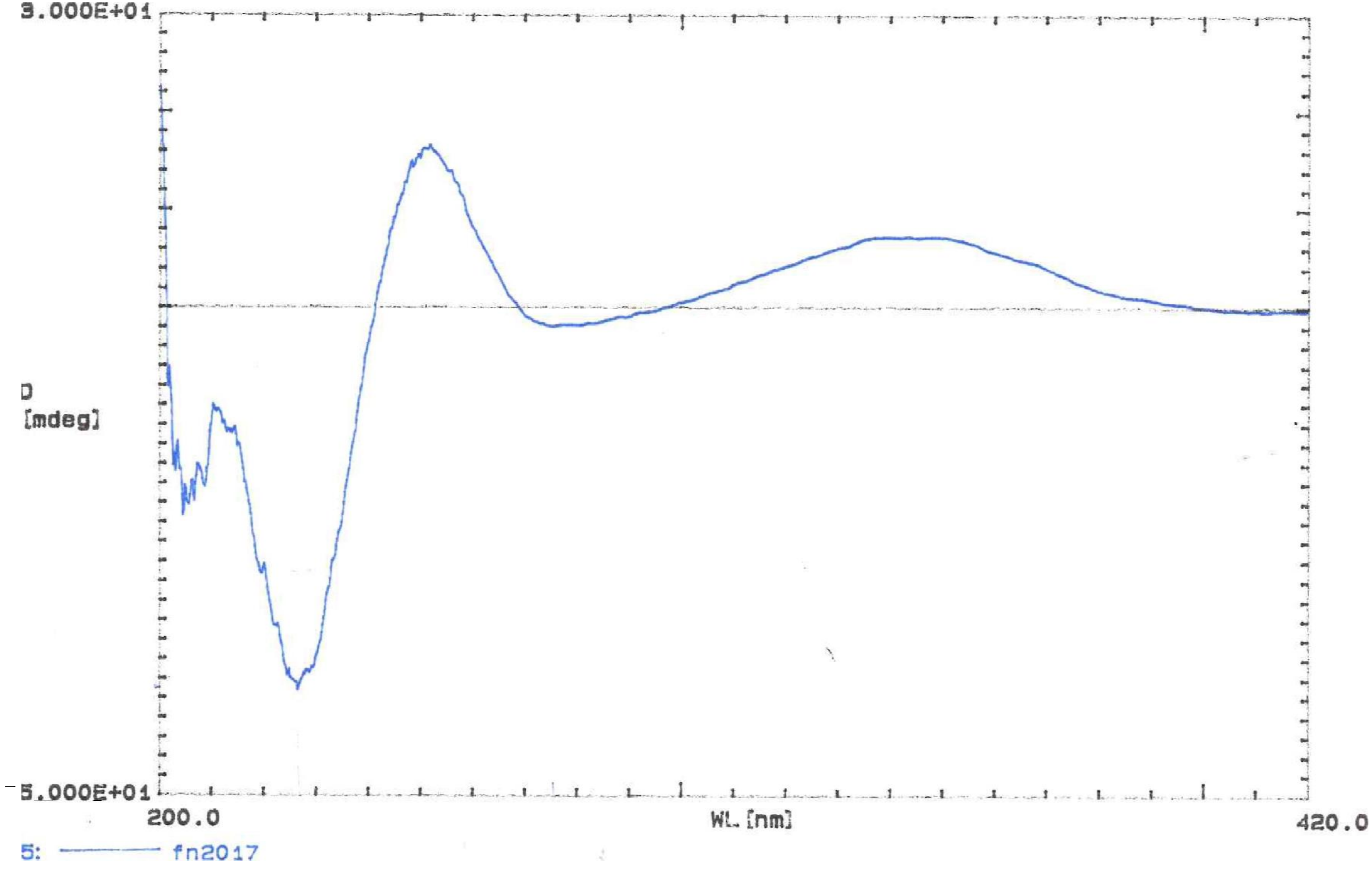

S22. CD spectrum of Tricholomalide F (28).

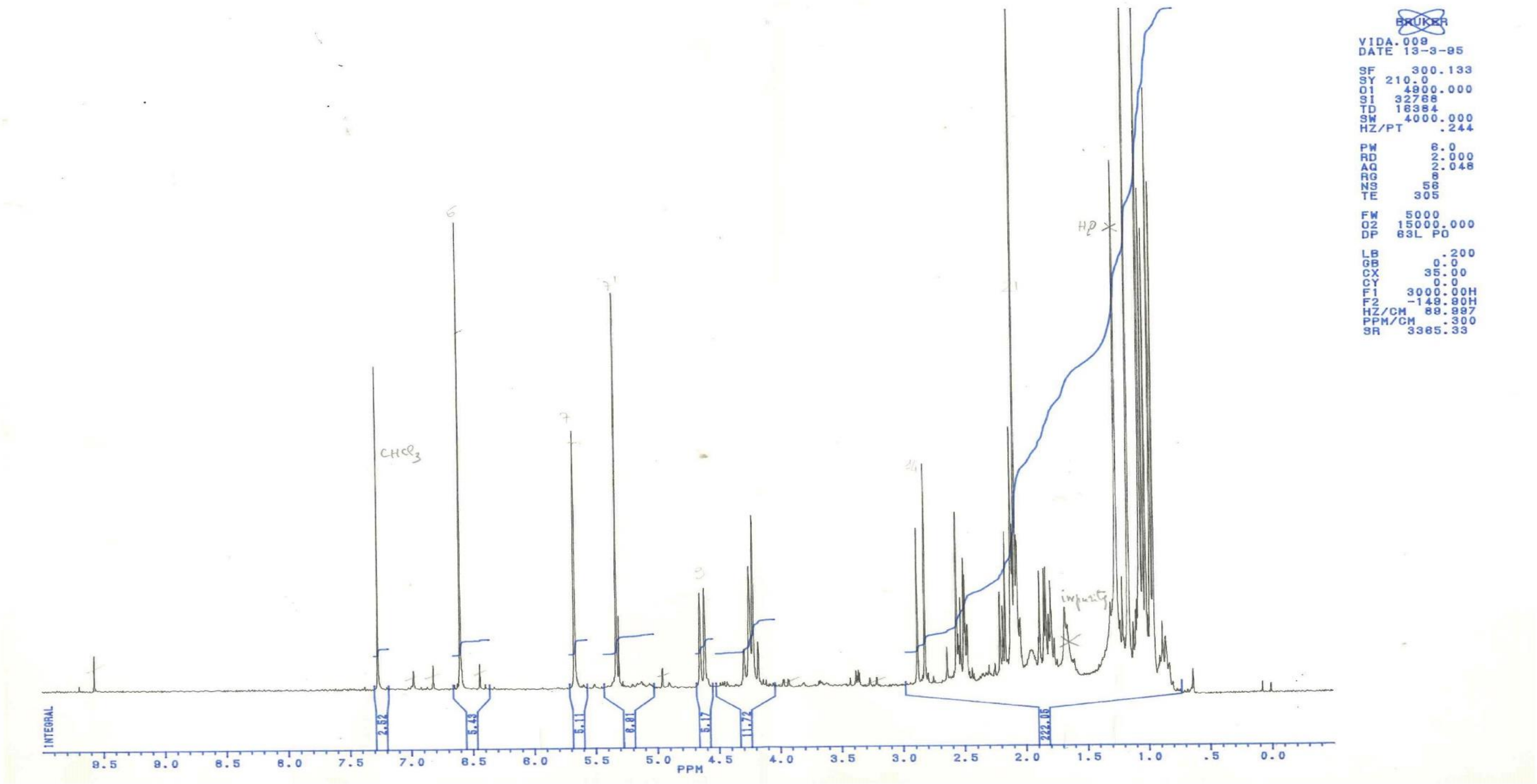

S23. ¹H NMR spectrum of Tricholomalide G (29).

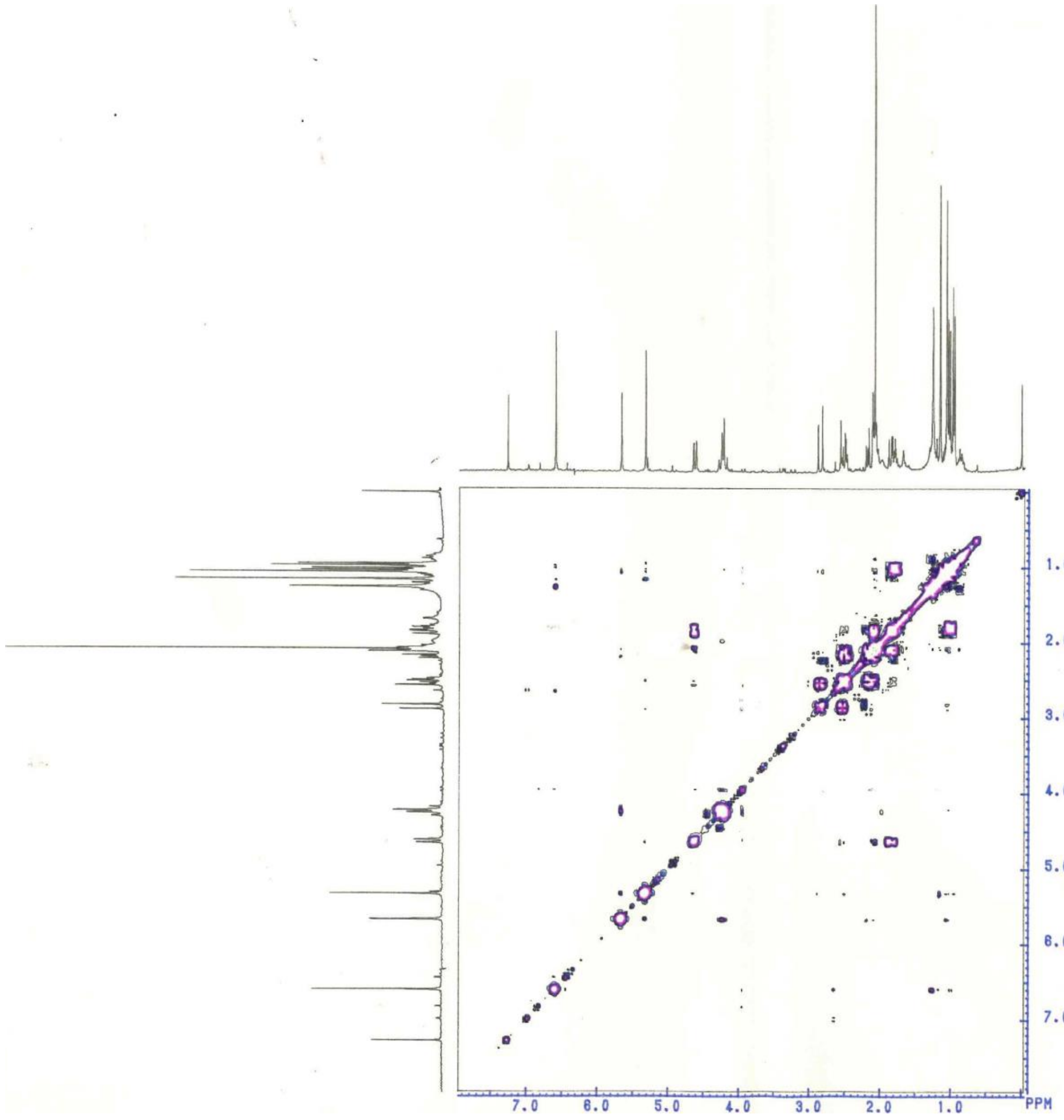

S24. COSY spectrum of Tricholomalide G (29).

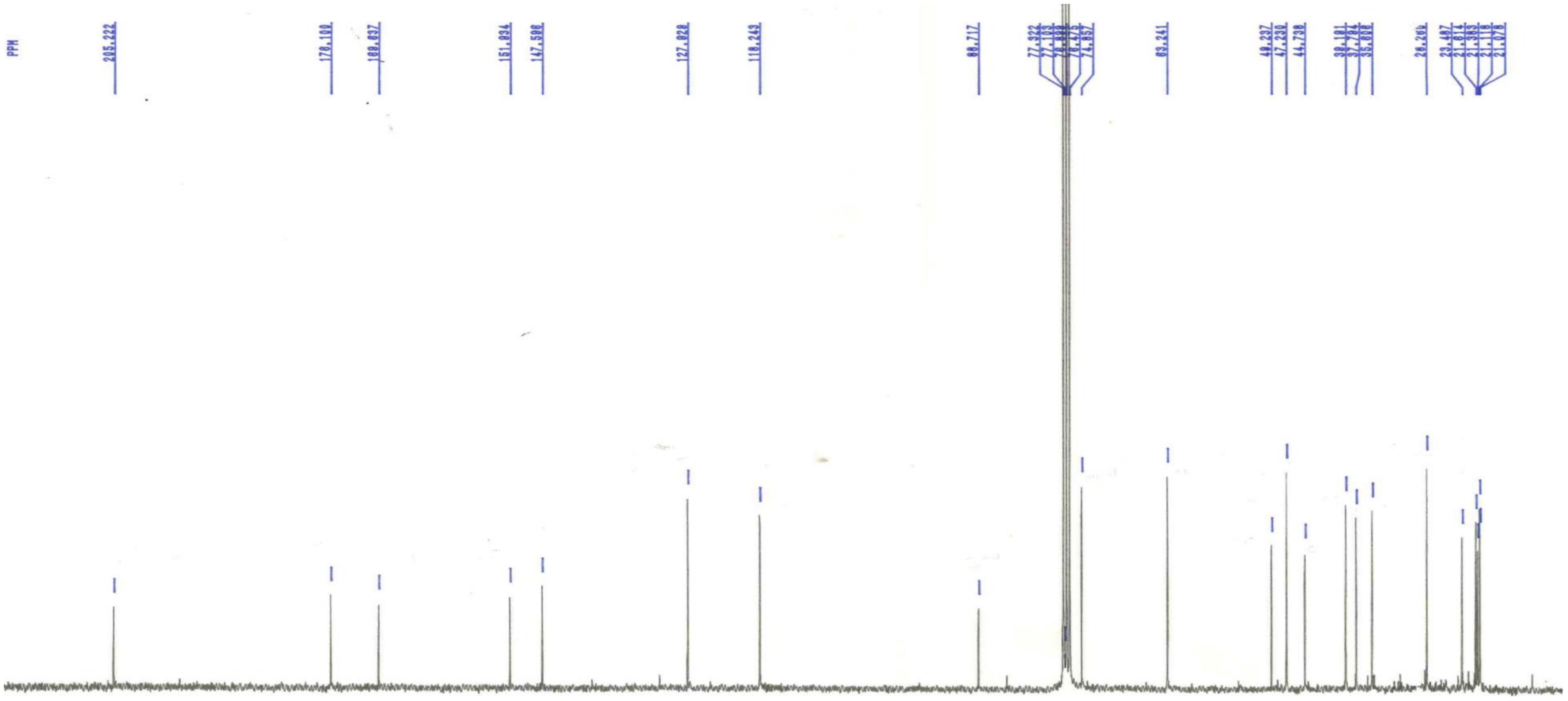

S25. ^{13}C NMR spectrum of Tricholomalide G (29).

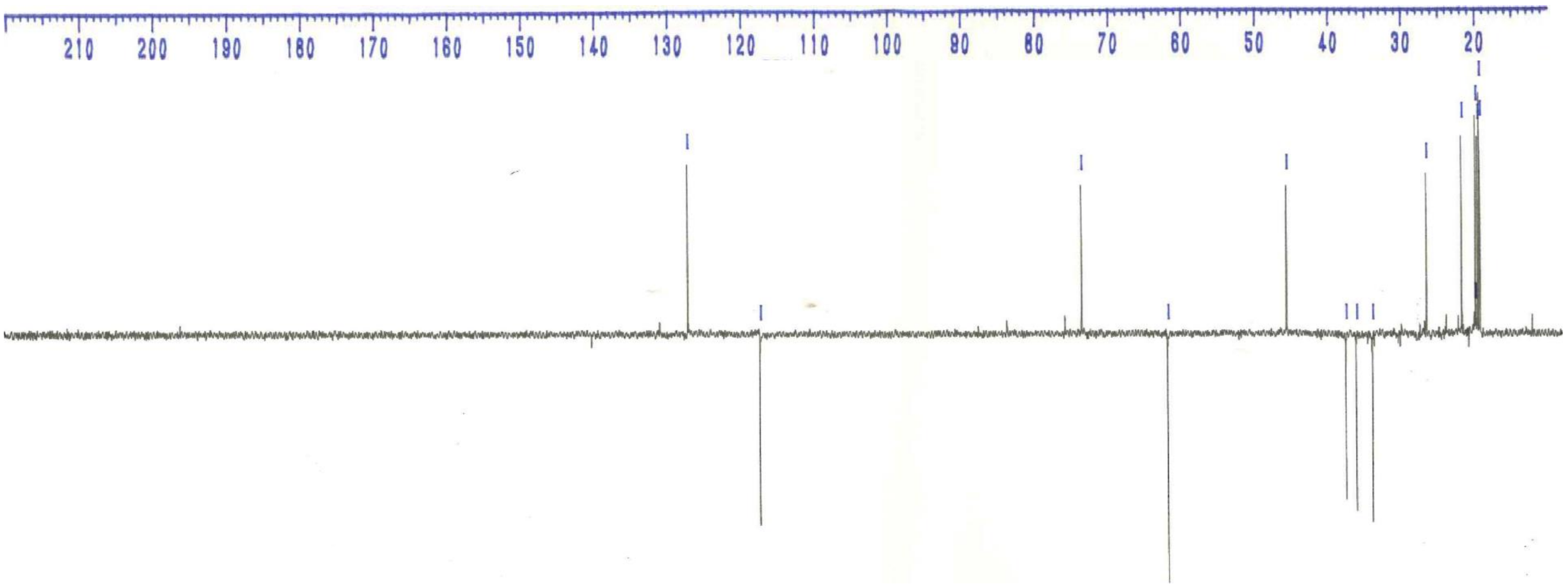

S26. DEPT ^{13}C NMR spectrum of Tricholomalide G (29).

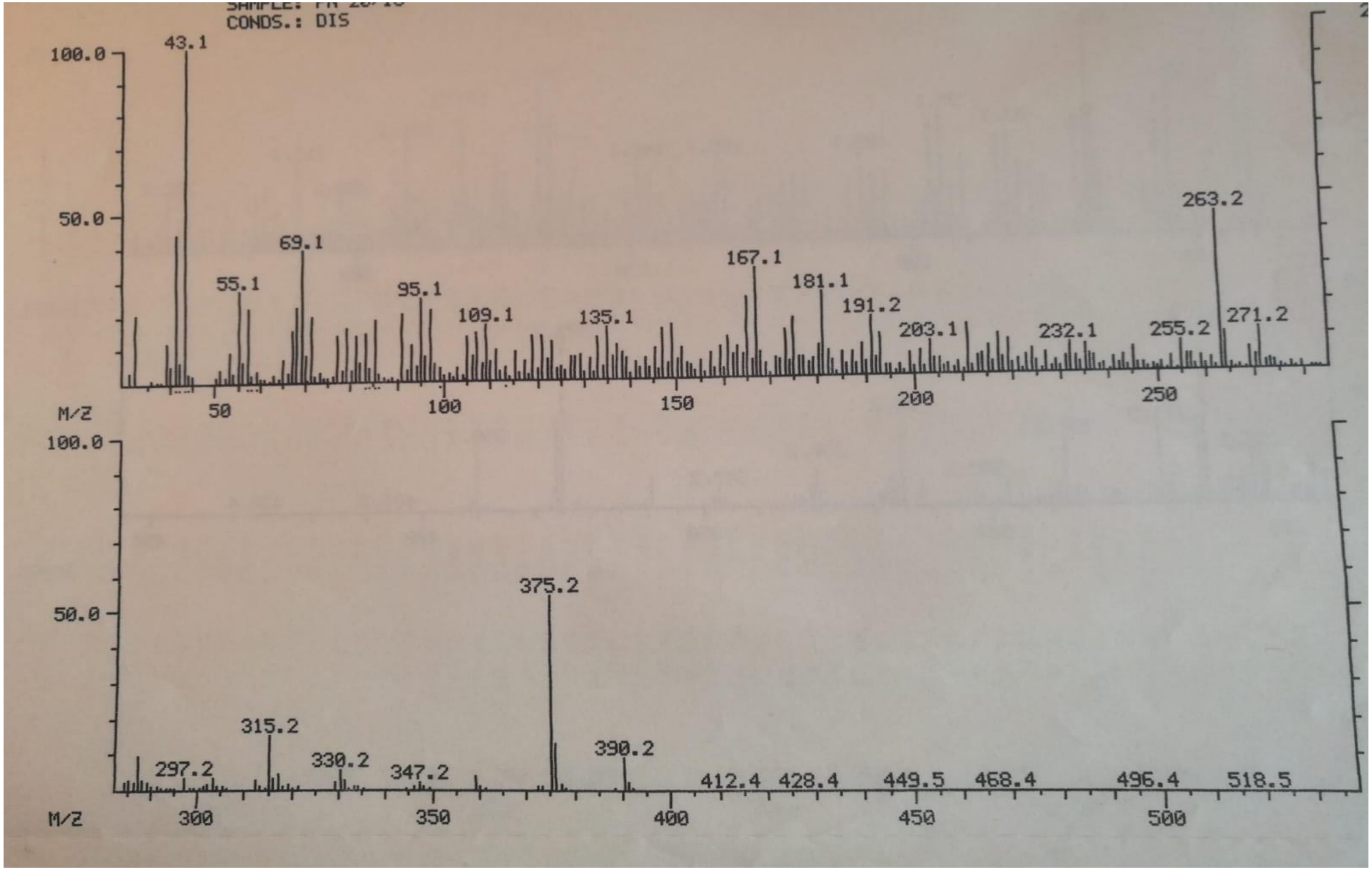

S27. EIMS spectrum of Tricholomalide G (29).

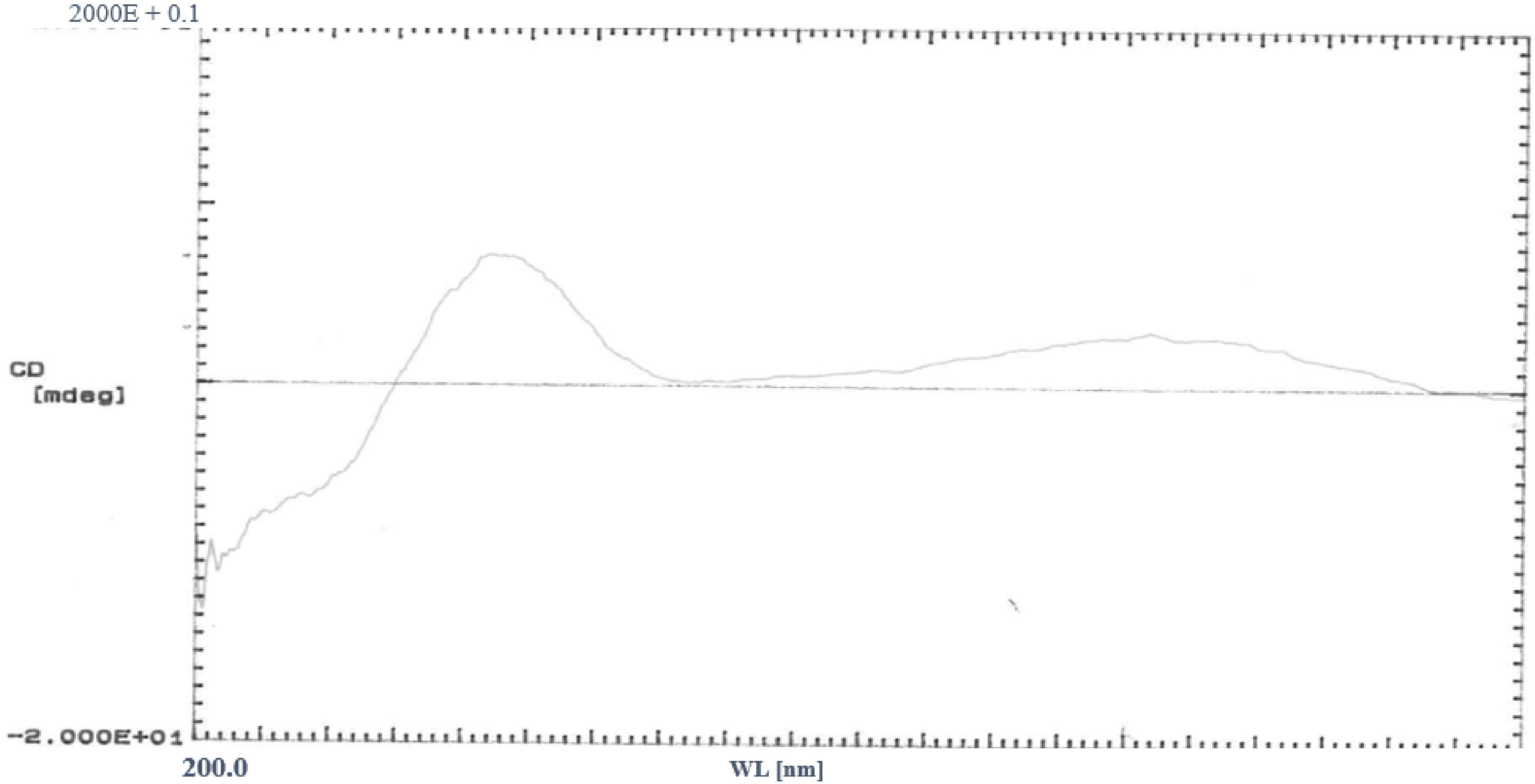

S28. CD spectrum of Tricholomalide G (29).

S29. Table S1. NMR spectral data for compounds **26**, **27**, and **29** in $CDCl_3$.

C/H	**26** δ_H [a,b]	**26** δ_C [c,d]	**27** δ_H [a,b]	**27** δ_C [c,d]	**29** δ_H [a,b]	**29** δ_C [c,d]
1	6.63 (1H, s)	125.9 d	6.65 (1H, s)	126.5 d	6.58 (1H, s)	128.0 d
2	–	87.4 s	–	87.4 s	–	88.8 s
3	–	148.6 s	–	148.8 s	–	147.7 s
4	9.70 (1H, s)	191.8, d	9.70 (1H, s)	192.2, d	4.22 (1H, dd, 13.0, 1.1)<br>4.27 (1H, br d, 13)	63.3 t
5	–	175.2 s	–	176.0 s	–	176.2 s
6	2.29 (1H, dd, 18.5, 1.0)<br>2.88 (1H, d, 18.5)	35.3 t	2.16 (1H, dd, 18.5, 1.0)<br>2.91 (1H, d, 18.5)	33.9 t	2.53 (1H, dd, 18.0, 1.0)<br>2.85 (1H, d, 18.0)	35.7 t
7	–	49.8 s	–	50.7 s	–	49.3 s
8	4.72 (1H, ddd, 11.5, 2.0, 1.0)	74.4 d	3.63 (1H, br d, 11.5)	71.6 d	4.63 (1H, ddd, 11.5, 2.0, 0.8)	75.0 d
9	1.79 (1H, dd, 15.0, 11.5)<br>2.15 (1H, dd, 15.0, 2.0)	37.7 t	1.85 (1H, dd, 15.5, 11.5)<br>2.15 (1H, dd, 15.5, 2.0)	41.1 t	1.84 (1H, dd, 15.5, 11.5)<br>2.02-2.20 (1H, m)	37.9 t
10	–	44.7 s	–	44.5 s	–	44.8 s
11	–	151.3 s	–	150.7 s	–	152.0 s
12	–	205.0 s	–	205.01 s	–	205.3 s
13	2.1-2.2 (1H, m)<br>2.45-2.57 (1H, m)	38.9 t	2.14 (1H, dd, 19, 12)<br>2.49 (1H, dd, 19, 8)	39.4 t	2.05-2.18 (1H, m)<br>2.47-2.57 (1H, m)	39.3 t
14	2.1-2.2 (1H, m)	47.2 d	1.85-1.92 (1H, m)	49.0 d	2.1-2.2 (1H, m)	47.3 d
15	1.75-1.85 (1H, m)	28.3 d	1.75-1.85 (1H, m)	28.6 d	1.75-1.88 (1H, m)	28.4 d
16	1.02 (3H, d, 6.5)	23.6 q	1.10 (3H, d, 6.5)	24.1[e] q	1.02 (3H, d, 6.5)	23.6 q
17	0.95 (3H, d, 6.5)	21.6 q	0.99 (3H, d, 6.5)	21.9 q	0.96 (3H, d, 6.5)	21.7 q
18	1.12 (3H, s)	21.4 q	1.09 (3H, s)	21.6 q	1.16 (3H, s)	21.5 q
19	0.98 (3H, s)	22.4 q	1.15 (3H, s)	22.7[e] q	1.06 (3H, s)	21.2 q
20	6.41 (1H, br s)<br>6.62 (1H, br s)	138.4 t	6.41 (1H, s)<br>6.63 (1H, s)	138.5 t	5.32 (1H, s)<br>5.67 (1H, t, 1.1)	118.3 t
COCH_3	–	169.4 s	–	–	–	169.7 s
CO***C***H_3	–	21.1 q	–	–	–	21.2 q
COC***H***$_3$	2.07 (3H, s)	–	–	–	2.08 (3H, s)	–

[a] 300MHz; [b] the chemical shift of the protons attached to each carbon was established by HETCOR cross peaks; [c] 75 MHz; [d] carbon multiplicities were established by DEPT experiments; [e] the assignments can be interchanged.

S30. Table S2. NMR spectral data for compound **28** in $CDCl_3$**.**

C/H	δ_H[a,b]	δ_C[c,d]	C/H	δ_H[a,b]	δ_C[c,d]
1	6.97 (1H, d, 1.0)	131.8 d	12	–	205.8 s
2	–	76.1 s	13	2.19 (1H, dd, 19.0, 12.5)<br>2.50 (1H, dd, 19.0, 8.0)	39.3 t
3	–	148.6 s	14	1.75–1.85 (1H, m)	47.5 d
4	9.55 (1H, s)	196.3 d	15	1.75-1.92 (1H, m)	28.6 d
5	–	175.3 s	16	1.11 (3H, q, 6.5)	24.1 q
6	β 1.86 (1H, d, 17.0)<br>α 3.24 (1H, dd, 17.0, 1.0)	35.5 t	17	0.98 (3H, q, 6.5)	21.8 q
7	–	48.6 s	18	1.20 (3H, s)	21.9 q
8	3.92 (1H, dd, 12.8, 2.5)	85.0 d	19	0.97 (3H, br s)	25.8 q
9	β 2.23 (1H, dd, 14.0, 2.5)<br>α 2.79 (1H, dd, 14.0, 12.8)	36.4 t	20	6.45 (1H, s)<br>6.82 (1H, s)	140.6 t
10	–	44.5 s	2′-O*H*	4.92 (1H, d, 1.0)	–
11	–	149.7 s			

[a]300MHz; [b]the chemical shift of the protons attached to each carbon was established by HETCOR cross peaks; [c]75 MHz; [d]carbon multiplicities were established by DEPT experiments.
